# Harnessing interpretable machine learning for holistic inverse design of origami

**DOI:** 10.1038/s41598-022-23875-6

**Published:** 2022-11-11

**Authors:** Yi Zhu, Evgueni T. Filipov

**Affiliations:** 1 Department of Civil and Environmental Engineering, University of Michigan, Ann Arbor, USA; 2 Department of Mechanical Engineering, University of Michigan, Ann Arbor, USA

**Keywords:** Mechanical engineering, Computer science

## Abstract

This work harnesses interpretable machine learning methods to address the challenging inverse design problem of origami-inspired systems. We established a work flow based on decision tree-random forest method to fit origami databases, containing both design features and functional performance, and to generate human-understandable decision rules for the inverse design of functional origami. First, the tree method is unique because it can handle complex interactions between categorical features and continuous features, allowing it to compare different origami patterns for a design. Second, this interpretable method can tackle multi-objective problems for designing functional origami with multiple and multi-physical performance targets. Finally, the method can extend existing shape-fitting algorithms for origami to consider non-geometrical performance. The proposed framework enables holistic inverse design of origami, considering both shape and function, to build novel reconfigurable structures for various applications such as metamaterials, deployable structures, soft robots, biomedical devices, and many more.

## Introduction

Origami, the art of folding paper, provides a method to build novel 3D engineering structures from flat 2D surfaces^1,2^. These origami structures can be used in a variety of applications such as biomedical devices^3,4^, micro/soft robots^5–7^, frequency selective surfaces^8,9^, metamaterials^10,11^, aerospace structures^12,13^, and many more. Over the years, there have been a number of inverse design methods proposed for origami systems^14–17^, but these methods and algorithms only solve kinematic design problems like fitting origami to arbitrary shapes and geometries. Designing functional origami structures for general engineering applications is still difficult because these active origami systems can have highly nonlinear motions, variable properties, and unintuitive multi-physical behaviors. Properly addressing a generic inverse design problem of origami requires considering the interaction between categorical and continuous features and handling formulations with multiple multi-physical objectives. These problems are difficult to handle with existing optimization-based inverse design methods for origami shape fitting so new solutions are needed.

Machine learning has proven to be a versatile and powerful method to solve physical science problems^18^, financial problems^19^, biomedical problems^20^, E-sport games^21^, etc. Moreover, a large number of different machine learning methods like neural network^22^, rule list^23^, boosting^24^, random forest^25^, and others have been developed to solve problems of different size, complexity, and nature. Because of the broadness and diversity of these methods, one key challenge on using machine learning is to select the appropriate method for a given problem. For origami type problems, machine learning techniques have been used to predict chaotic dynamic responses^26^ and to solve for origami folding motions^27^. In addition, interpretable machine learning techniques were also used for handling design problems for metamaterials systems that have different input data types when compared to origami^28^. However, no prior work has tackled the inverse design problem for origami using interpretable machine learning techniques.

**Figure 1:**
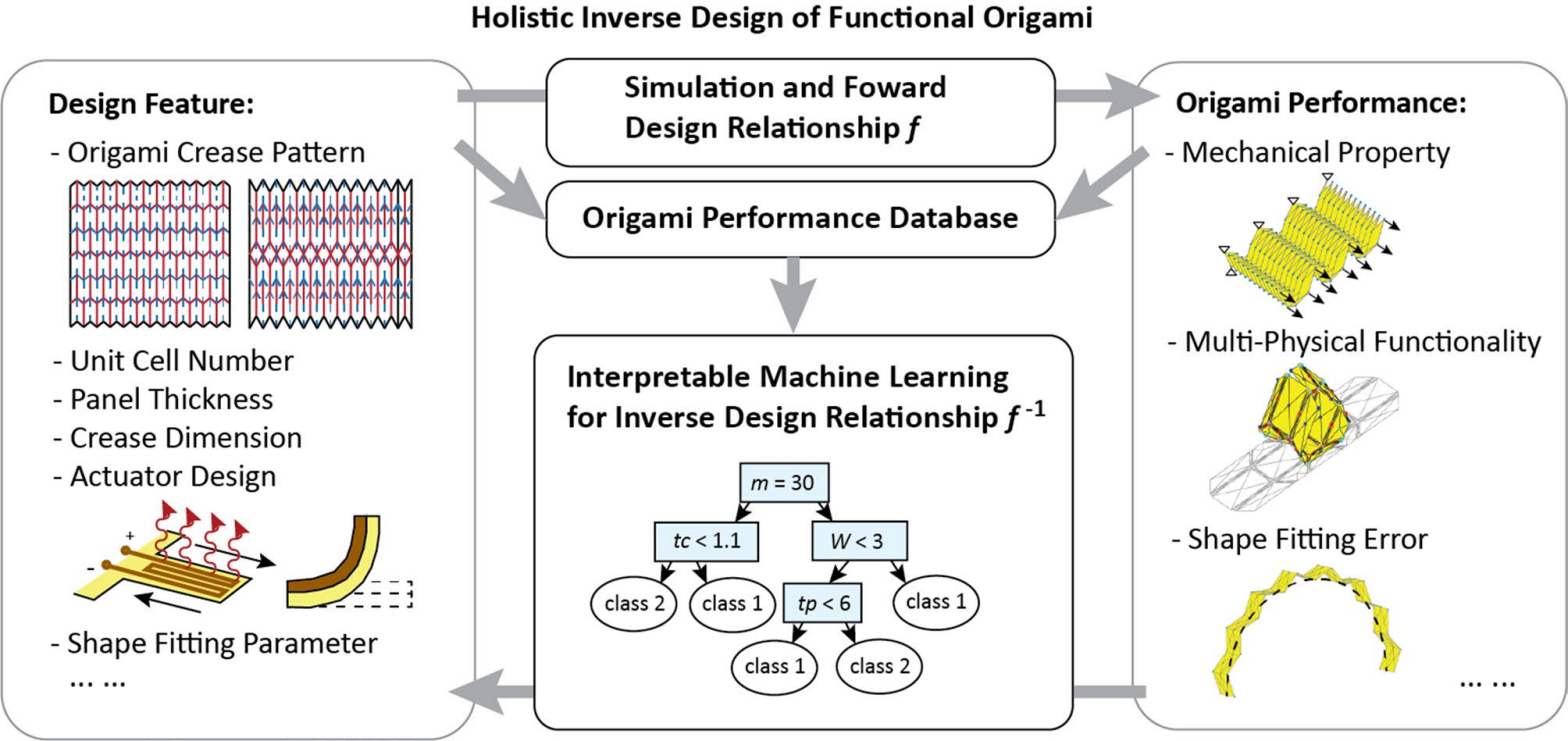
Interpretable machine learning for inverse design of origami. The relationship between origami design features (left) and performance of origami systems (right) can be thought of as a black-box nonlinear function $f$. This work shows that it is possible to train an interpretable machine learning method (a decision tree-random forest method at the bottom) to uncover the underlying structure of this black-box nonlinear function $f$, so that we can build human understandable design rules to solve the inverse design problem (solve for ${f}^{-1}$).

In this work, we show that an interpretable machine learning method called the decision tree method and its ensemble version called random forest^25,29^ are particularly suitable for the inverse design of functional origami. Figure 1 summarizes the fundamental idea of this work. The design of origami can be thought of as building a nonlinear function $f$ to calculate the *performance indices* of the system (such as stiffness, Poisson’s ratio, material cost, etc.) based on given *design features* (such as the number of origami cells, the thickness of materials, sector angles of the origami pattern, etc.). Usually, numerical simulations are used to represent this function $f$ because it is too complex to be expressed in a closed form. In a traditional setting, designing origami structures is accomplished through plotting and observing the relationships between the features and the performance using nonlinear simulation methods^30,31^. To extend this traditional design method to an inverse design setup, one needs to construct an *inverse relationship* ${f}^{-1}$ that calculates a set of design features from the target performance. In this work, we show that it is possible to compute this inverse relationship ${f}^{-1}$ by first populating an origami performance database and then applying interpretable machine learning to fit the database (approximating $f$). Unlike standard “black box” machine learning methods, interpretable machine learning can produce human-understandable decision rules with which people can understand why certain judgments are made by the algorithms^32,33^. Once an *interpretable* approximation of the nonlinear function $f$ is obtained, the inverse relationship ${f}^{-1}$ can be constructed. Through our implementation and exploration of the decision tree method for functional origami systems, we found that selecting the “more informative” branches provides useful design rules for this set of problems (finding the inverse relationship ${f}^{-1}$).

In the following sections, we demonstrate how to resolve the origami inverse design problem as a data science and machine learning problem. We show how to build the origami performance database and use the decision tree-random forest method to compute human-understandable decision rules for the inverse design of functional origami. First, we demonstrate the methodology using a simple design problem on Miura origami cells. Next, we show that the proposed method can handle categorical features and compare different origami patterns for a more complex origami metasheet design problem. We then show that the tree-based method can tackle multi-objective and multi-physical problems by designing a set of electro-thermal origami grippers. Finally, with a design problem on origami arches, we demonstrate that the proposed method can enable origami shape fitting algorithms to further consider non-geometrical properties so that holistic origami design can be achieved. These examples show that the decision tree-random forest method offers unprecedented versatility for the inverse design of origami systems.

## Results

### Computing origami design rules with interpretable machine learning

**Figure 2:**
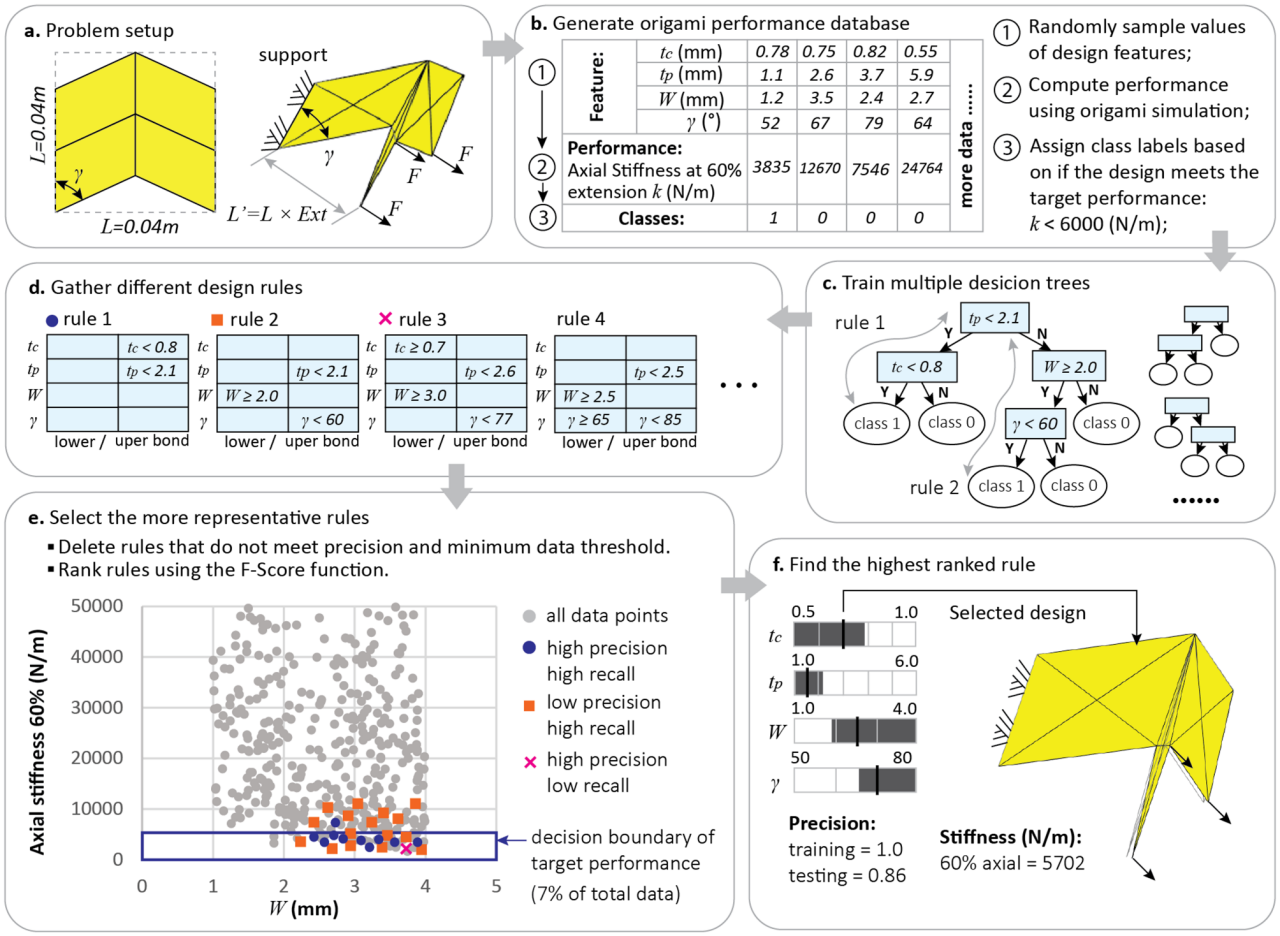
Computing interpretable design rules for origami assemblages. (**a**) The problem setup for the single unit Miura-ori cell. The axial stiffness is computed for the folded origami with supports on the left and axial loads on the right. (**b**) A database of Miura-ori design features and resulting axial stiffness performance is populated using a physics-based origami simulator. Data points are labeled based on whether they meet the target performance or not (axial stiffness *k* < 6000 N/m). (**c**) A number of decision trees are trained to classify the database. (**d**) Design rules are gathered by collecting the splitting criteria in each branch of the decision tree (following the gray lines in the sub-figure (C)). (**e**) Representative rules are selected based on the precision, the recall, and the number of data points satisfying the rules. In this case, the rule 1 (blue dot) is better than the rule 2 (orange box) and the rule 3 (pink cross) because it has high precision and recall. (**f**) A final design rule with the highest F-score is picked. The box chart contains the full range for all features, while the shaded regions indicate the final design rule for the target performance. A Miura-ori design that follows the final rule is shown (indicated with dark lines in the box chart).

This section introduces how the decision tree-random forest method can be used to compute design rules for the inverse design of origami systems. To demonstrate the methodology, we use a simple design problem on Miura-ori unit cells as presented in Fig. 2a. The design objective is to find a Miura-ori unit cell to have an axial stiffness $k$ smaller than $6000 \, {\text{N/m}}$ (target performance: $k<6000 \, {\text{N/m}}$). The Miura-ori cell is cut from a square sheet and is folded to a 60% extension ratio, defined using the ratio between the folded length and flat length of the pattern ($Ext=L{^{\prime}}/L=60 \%$). The axial stiffness is calculated by fixing the unit cell at one end and applying small forces to stretch it at the other end. For this problem, four design variables (features) are used including the thickness of panels (${t}_{p}$), the thickness of creases (${t}_{c}$), the width of creases ($W$), and the sector angle of the Miura-ori pattern ($\gamma$). We determine the design ranges for these features based on practical fabrication and material limits, and they are: $1.0 \, {\text{mm}}<{t}_{p}<6.0 \, {\text{mm}}$, $0.5 \, {\text{mm}}< {t}_{c}<1.0 \, {\text{mm}}$, $1.0 \, {\text{mm}}<W< 4.0 \, {\text{mm}}$, and $50^\circ <\gamma <80^\circ$.

To build a database for training the machine learning model, we randomly create designs using different feature values within the specified ranges (step 1 in Fig. 2b). Each column of the table in Fig. 2b represents one design (data point) and 1000 random designs are generated for this example problem. Next, we compute the axial stiffness (or the performance) for each data point using a physics-based origami simulation package called SWOMPS^34^ (step 2 in Fig. 2b). The SWOMPS package is used in this paper because it is computationally efficient and can capture multi-physical behaviors important for many origami designs^35,36^ (see Materials and Methods). This simulation package is based on a bar and hinge mechanics representation of origami systems and contains a reduced-order model for electro-thermally actuated creases. The simulation results of this package have been verified against both FE simulations and physical experiments^35,36^ so the generated database is representative of realistic behaviors in active origami structures. Based on the simulated performance of each data point, we assign class labels depending on if the design can achieve the target performance or not (step 3 in Fig. 2b). Data points that meet the target ($k<6000 \, {\text{N/m}}$) are assigned a label of 1 while the remaining samples are assigned a label of 0.

Next, we use the design features and the class labels to set up a supervised learning problem that can be solved using the decision tree-random forest method. The method is trained to differentiate designs that meet the target performance (with label 1) from those that do not (with label 0) based on the design feature values. Figure 2c shows a sample decision tree for the design problem of the Miura-ori unit cell. To determine the class of a data point, we send the data point into the tree from the top. Each time the data point encounters a branch node, a simple criterion is checked to determine if the point should go to the left branch or to the right branch. For example, a datapoint with ${t}_{p}=1.1 \, {\text{mm}}$ at the first node of the sample decision tree (shown on Fig. 2c) will be sent to the left branch because ${t}_{p}=1.1 \, {\text{mm}} <2.1 \, {\text{mm}}$. After a series of judgments, the data point will be sent to a leaf node, where no more branching occurs, and a class label is predicted. For example, the datapoint listed in column one of the Table in Fig. 2b will follow the gray arrow (marked as “Rule 1”) downward and be judged as a Class 1 data. This means the machine learning algorithm *thinks* that this feature design (${t}_{c}=0.78 \, {\text{mm}}$, ${t}_{p}=1.1 \, {\text{mm}}$, $W=1.2 \, {\text{mm}}$, $\gamma =52^\circ$) is most likely to produce a single unit Miura-ori with axial stiffness $k<6000 \, {\text{ N/m}}$. The algorithm comes to this conclusion because the feature design values match the rule: ${t}_{c}<0.8 \, {\text{mm}}$, and ${t}_{p}<2.1 \, {\text{mm}}$. As such, each branch associated with Class 1 in the decision tree gives a design rule that would produce an origami design that meets the target performance. This highly interpretable structure of a tree method is useful for inverse design because the inverse relationship (i.e. ${f}^{-1}$) shows how to pick features based on the target performance. Moreover, we use randomly selected sub-datasets to train different decision trees to create more potential design rules. Training multiple decision trees forms an ensemble version of the tree method called a *random forest*. The structure of the trees, the splitting criteria, and the leaf node predictions are learned by the machine learning algorithm during the training process using a machine learning package called sklearn^37^. After training multiple decision trees, we gather all the design rules by tracing back through the tree branches (Fig. 2d). For example, Rule 1 and Rule 2 gathered in Fig. 2d are correlated to the two different branches in the sample tree (gray arrows in Fig. 2c).

Although these decision trees are automatically learned by the machine learning method, there are other manually specified variables that control how decision trees are computed. These user-specified variables are referred to as hyperparameters in machine learning and a technique called grid search is usually performed to select these hyperparameters. Different combinations of the hyperparameters are used to train the machine learning algorithms and the best combination is selected. The hyperparameters considered in this work includes: (i) the maximum depth of the trees, (ii) the number of tree learners in the forest, (iii) the cost-complexity pruning alpha value, (iv) the splitting criterion, and (v) the sub-dataset ratio for training different decision trees. Details on the considered hyperparameters and the grid search are provided in the Supplementary Materials Section S3.3.

Now that we have collected a number of potential design rules, we need to select those that provide *better* performance for the inverse design problem. More specifically, a better design rule needs to have a higher *precision* value and a higher *recall* value, where a high precision means that the rule is accurate, and a high recall means that the rule is representative (see the method section for details). In this work, we use the following routine to select design rules with better performance. The rules need to satisfy two thresholds and they are: (1) the precision is greater than 0.9, and (2) the number of data points satisfying the rule is greater than 10. Rules that do not satisfy these thresholds are eliminated from further consideration. Next, we rank the rules using the F-score function^38^:

$${F}_{\beta }=\left(1+{\beta }^{2}\right)\left(\frac{\mathrm{precision}\cdot \mathrm{recall}}{{\beta }^{2}\cdot \mathrm{precision}+\mathrm{recall}}\right)$$

This F-score function creates an average score from both the precision and the recall value, where the recall is seen as $\beta$ times more significant than the precision value. In this work, we select a value of $\beta =0.2$ because the precision is more important for an inverse design problem, where the goal is to find “some designs” that meet the target performance not “all designs” that meet the target performance. Finally, we select the rules with the highest F-scores for our origami design and Fig. 2f shows the selected rule for this demonstration example. The shaded (darker) region of the box chart indicates the computed range of the four design variables and the design rule is ${t}_{c}<0.78 \, {\text{mm}}$, ${t}_{p}<2.2 \, {\text{mm}}$, $W\ge 1.9 \, {\text{mm}}$, $\gamma \ge 67^\circ$.

Although we managed to find a rule that performs well in the training dataset with a precision of 1.0, we need to further test the rule to verify that it is indeed a good rule. The testing is conducted by computing the precision of the rule using another testing dataset that is not used for training the machine learning method. This process is usually referred to as the *hold-out* testing in machine learning. Basically, the training data are homework problems for the machine learning algorithm and the testing data is the final exam. Details of the hold-out testing setup can be found in the Supplementary Materials Section S3.3. In this demonstration example, a testing precision of 0.86 is obtained, which is reasonably good for an unbalanced dataset like the one for the single unit Miura cells, where the target data only consists of 7% of the total dataset.

Once good design rules are obtained and their quality is confirmed through testing, a designer can directly use these rules to design a suitable origami structure. Figure 2f presents one sample design that satisfies the rules and has the following features: ${t}_{c}=0.70 \, {\text{mm}}$, ${t}_{p}=1.5 \, {\text{mm}}$, $W=2.5 \, {\text{mm}}$, $\gamma =70^\circ$. This design has an axial stiffness $k=5702 \, {\text{N/m}}$, which indeed meets the target performance.

### Comparing different origami patterns

It is difficult to inverse design functional origami considering multiple origami patterns because *categorical features* are needed to represent and compare these different patterns. Categorical features including the type of origami pattern, the number of unit cells, and the topological design of each pattern, cannot be implemented directly into common continuous optimization-based inverse design methods^14,15^. Capturing and comparing different patterns is essential because different origami produce intrinsically different motions and functional performances. To address this challenge, this section shows that the decision tree-random forest method can handle the complex interaction between categorical and continuous variables, which allows the method to compare and select between different origami patterns.

**Figure 3:**
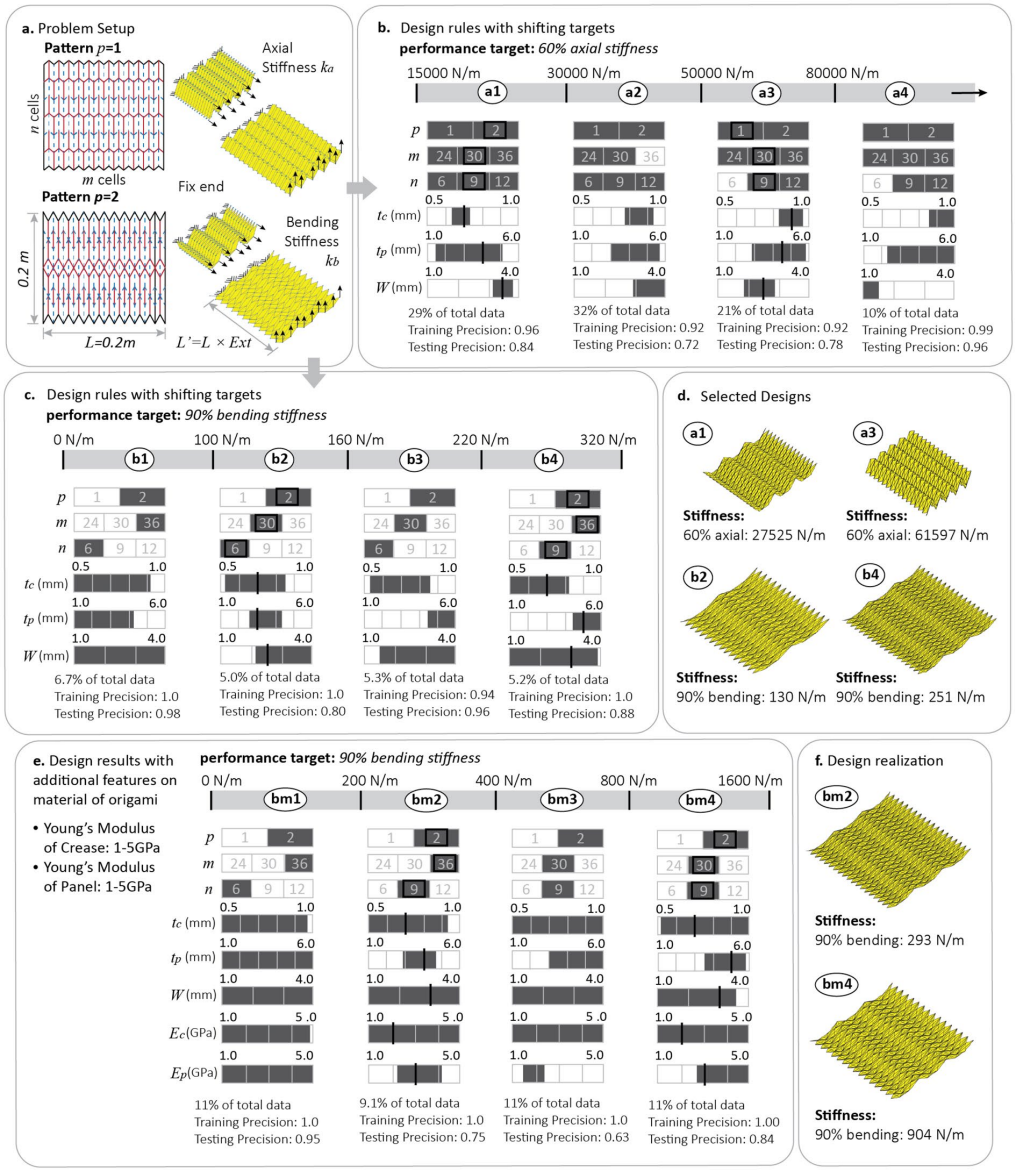
Comparing different origami patterns using the decision tree-random forest method. (**a**) Problem setup: two origami patterns are compared for their performance as a metasheet. (**b**) Design rules for different axial stiffness targets when the metasheet is at a 60% extension. (**c**) Design rules for the bending stiffness at a 90% extension. (**d**) Sample designs for the four selected design targets. (**e**) Design rules after adding design features regarding the materials stiffness. (**f**) Design realizations of the origami metasheet after adding materials stiffness as design features.

Here, we study a design problem for origami metasheets shown in Fig. 3a. These origami metasheets are cut out from a thin square plate with a footprint of $0.2 \, {\text{m}} \times 0.2 \, {\text{m}}$ and can be built from two distinctly different origami patterns including the standard Miura-ori pattern (Pattern 1) and the Tachi-Miura Polyhedron (TMP)^30^ (Pattern 2). To represent these two patterns, we introduce an integer (binary) variable $p = \{\mathrm{1,2}\}$. In addition, these origami metasheets can have different numbers of unit cells, represented as $m=\{24, 30, 36\}$ and $n=\{6, 9, 12\}$, in the two directions. In addition to these three categorical features, three continuous design features are also used in this problem, and they are the thickness of panels ($1.0 \, {\text{mm}}<{t}_{p}<6.0\, {\text{ mm}}$), the thickness of creases ($0.5 \, {\text{mm}}<{t}_{c}<1.0 \, {\text{mm}}$), and the width of creases ($1.0 \, {\text{mm}}<W<4.0 \, {\text{mm}}$). An origami database of 2000 Miura origami samples and 2000 TMP origami samples is populated by randomly sampling values of the other design features. The origami simulation package SWOMPS is used to calculate the stiffness performance of the different origami systems (details in the Supplementary Materials Section S2). In this example, we separately design the metasheet for two targets including the axial stiffness ${k}_{a}$ at 60% extension and the bending stiffness ${k}_{b}$ at 90% extension (design for multiple targets/objectives is discussed in the next section). As before, we define the extension of the origami ($Ext$) as the ratio between the folded length to the flat length. Four stiffness target zones are created for both the axial stiffness and the bending stiffness as indicated in Fig. 3b and c.

We first study the design for axial stiffness ${k}_{a}$ at 60% extension (see Fig. 3b). Assume that we want to design an origami metasheet to have $15,000 \, {\text{N/m}}<{k}_{a}<30,000 \, {\text{N/m}}$ (Zone a1), we can label data that meet the target as 1 and the rest of the data as 0. After labeling, we can use the procedure in Fig. 2 to compute the design rules for this target. The design rule with the best performance is shown in the left column of Fig. 3b. We can then repeat the process for the other three target zones (Zone a2 to a4) by reusing the existing database. The computed rules for all targets are shown in Fig. 3b, and this series of rules tells us how the preferred design for an origami metasheet changes as the target for axial stiffness is increased. Interestingly, the machine learning method prefers changing the continuous variables to achieve the different axial stiffness targets without paying much attention to the categorical features used to represent different origami patterns. More specifically, the machine learning method suggests that controlling the thickness of creases (${t}_{c}$) and the width of the creases ($W$) are more important than other parameters because tighter thresholds are used for these two features. Two sample metasheet designs for target Zones a1 and a3 are shown in Fig. 3d. The resulting performance of these designs indeed falls within the desired targets.

However, the design rules can be very different when we study the bending stiffness at 90% extension. Similarly, four design rules are computed for four different target zones as shown in Fig. 3c. Here, we set the targets to contain less data points in order to test out how well the proposed method performs when dealing with unbalanced databases, where the targets only contain around 5% of the total data. When we investigate the result of this series of design rules, we see that the machine learning method is paying more attention to the categorical features. As the target moves from one zone to another, the computed design rules change in a non-continuous manner because of the complex interactions between categorical variables and continuous variables. For example, when the target changes from Zone b2 to Zone b3 where we have a stiffer target, the machine learning method indicates that increasing the thickness of the panel is sufficient to meet the target. However, when we further increase bending stiffness requirement as we move from Zone b3 to Zone b4, the method suggests that it is better to change the categorical features (number of cells) and the continuous features simultaneously. A similar categorical jump is also observed when the target moves from Zone b1 to Zone b2. The proposed method can capture these complex interactions between the continuous features and categorical features, which cannot be done with optimization-based design methods.

To consider the influence of material properties on the inverse design problem, we add two design features representing the Young’s moduli of creases and panels. The inverse design results with this new database are demonstrated in Fig. 3e and f. The results show that the proposed methodology can still handle the problem relatively well after adding in the new material design features, and that there are still complex interactions between categorical and continuous features like those we found in Fig. 3c. Adding the material properties does not change the underlying mathematical formulations of this inverse design framework, so the proposed methodology is still applicable to the problem.

## Multi-objective design for multi-physical functional origami

**Figure 4:**
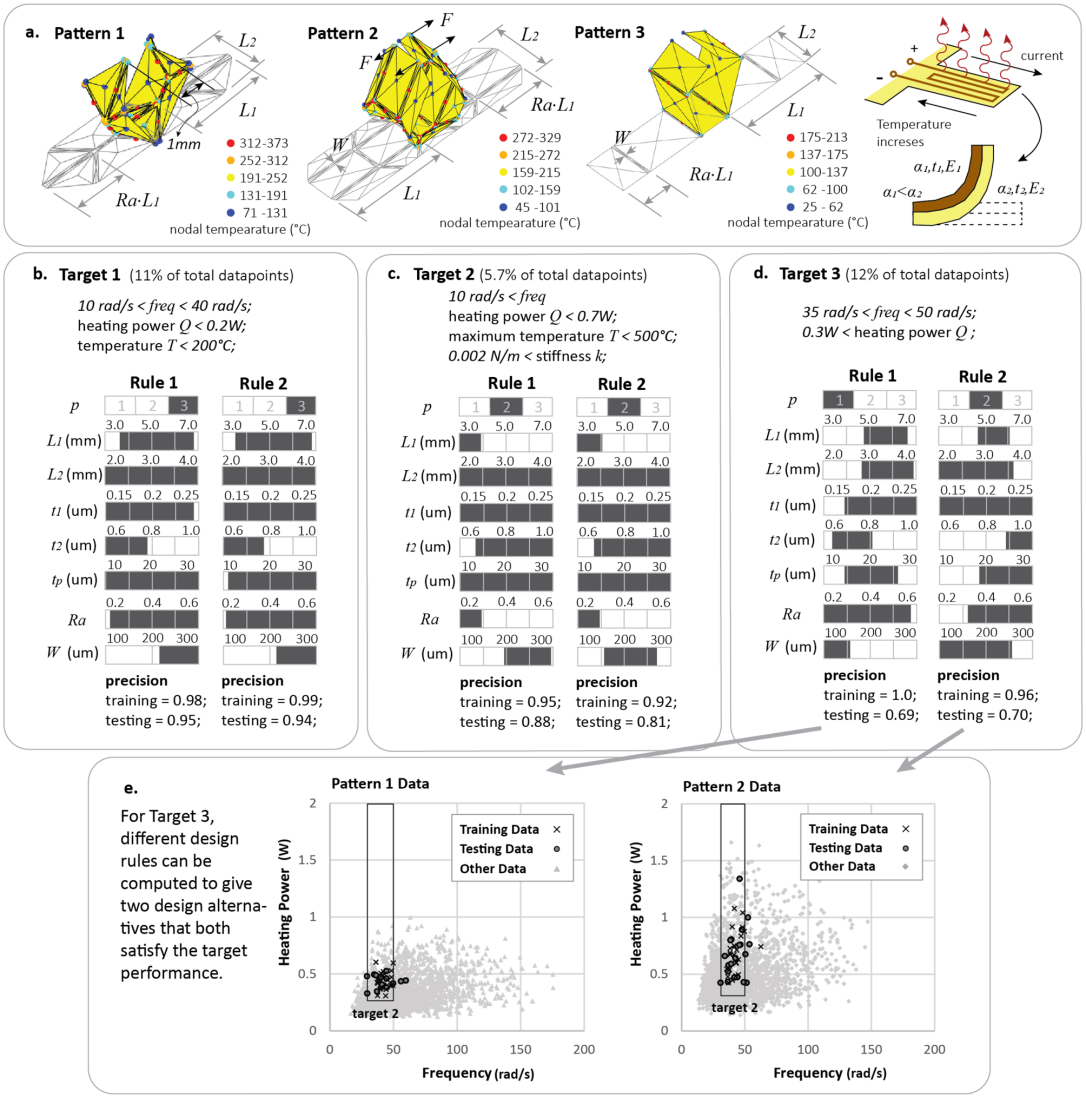
Design of an origami gripper with multi-physics and multiple objectives. (**a**) Problem setup for building active electro-thermal origami grippers with three different patterns. (**b**–**d**) The top two design rules of designing the grippers for three different multi-objective targets (Targets 1 to 3). (**e**) The top two design rules for Target 3 give distinct design alternatives where a different origami pattern is selected, yet both data point distributions meet the given target.

Handling multiple objectives is often necessary for designing functional origami structures because these systems exhibit multi-physical behaviors that need to be measured and compared using several different indices. Because such multi-objective problems are difficult to handle with standard optimization-based techniques, most existing functional origami systems were designed using trial-and-error approaches^4–6^. In this section, however, we demonstrate that the tree method can effectively handle multi-physical behaviors and can simultaneously consider multiple objectives. To this end, we present an example design for an electro-thermally actuated origami gripper where dynamics, power consumption, thermal behavior, and stiffness are all of interest (see Fig. 4).

In this example, one of three origami gripper patterns can be selected to achieve the target gripping motion (closing the gripping tip to less than a 1 mm gap). We assume that the gripper is actuated at the creases using an electro-thermal bi-layer system demonstrated in Fig. 4a and discussed in detail in^7^. This actuator contains two material layers with different coefficients of thermal expansion where one layer also serves as an electro-thermal heater (the top layer in this case). Joule heating causes differential expansion in the two layers, local curvature at the crease, and global folding of the origami patterns. Design features for the gripper include one categorical variable $p$ used to describe the pattern, and seven continuous variables including the length (${3.0 \, {\text{mm}}<L}_{1}<7.0 \, {\text{mm}}$) and width ($2.0 \, {\text{mm}}<{L}_{2}<4.0 \, {\text{mm}}$) of the gripper arm, the location of the first hinge $(0.2<Ra<0.6)$, the width of the actuator creases ($100 \, \upmu {\text{m}}<W<300 \, {\text{m}}$), the thickness of the two layers in the actuator design ($0.15 \, \upmu {\text{m}}{<t}_{1}<0.25 \, \upmu {\text{m}}$ and $0.6\, \upmu {\text{m}}<{t}_{2}<1.0 \, \upmu {\text{m}}$), and the thickness of the panels 10 ($\, \upmu {\text{m}}<{t}_{p}<30 \, \upmu {\text{m}}$).

We use four indices to compare the multi-physical performance of the gripper, specifically (1) the fundamental frequency ($freq$) of the gripper, (2) input heating power ($Q$) needed to close the gripping arm, (3) maximum crease temperature ($T$) during the gripping motion, and (4) stiffness ($k$) of the gripper in resisting loads applied to pry it open. The origami simulation package SWOMPS is used to simulate and find these four performance indices (see Supplementary Materials S2 for details).

To demonstrate how interpretable machine leaning can tackle multiple multi-physical objectives, suppose we want to design an origami gripper to simultaneously match the following performance indices: $10 \, {\text{rad/s}}<freq<40 \, {\text{rad/s}}$, heating power $Q<0.2 \, {\text{W}}$, and maximum temperature $T<200 \,^\circ {\text{C}}$ (Target 1). We label all data points that satisfy the performance targets to be Class 1 and label the rest of the data as Class 0. Then, by computing the more representative decision rules for Class 1, we obtain design rules for a functional origami that will satisfy all three performance indices simultaneously. Figure 4b shows two design rules that have the highest F-score for this Target 1. Interestingly, both rules have high precision and are nearly identical except for small differences in the selection of ${t}_{1}$ and ${t}_{p}$.

We can use the same method to simultaneously design for all four performance indices (Target 2: frequency $10 \, {\text{rad/s}}<\, {\text{freq}}$, heating power $Q<0.7$ W, and maximum temperature $T<500 \, ^\circ {\text{C}}$, and stiffness $0.002 \, {\text{N/m}}<k$), with the result presented in Fig. 4c. If we compare the computed rules for Target 2 with those for Target 1, we can see that the machine learning method has picked another pattern after adding in the minimum prying stiffness requirement. While Pattern 3 was selected for Target 1, its horizontal creases cannot provide additional stiffness to resist prying, so Pattern 2 is now selected to achieve Target 2. This high interpretability of the tree methods helps users to better understand and reason about the desired behaviors of functional origami systems. Moreover, the machine learning method also shows how important each feature is for different design targets. For example, controlling the values of the gripping arm length ${L}_{1}$ and the location of the first creases (defined by $Ra$) is only important for Target 2 but not for Target 1.

In general, the design rules with the highest F-scores obtained from the machine learning method tend to be similar to each other. However, Fig. 3d shows an interesting result where the top two competing rules have relatively large differences between them. The large difference is because the two rules select different origami patterns, and the machine learning method thinks that both of these rules are appropriate for the desired design. Rule 1 of Target 3 selects Pattern 1 while Rule 2 selects Pattern 2. These results highlight that by computing multiple rules with high F-scores, it is possible to find *distinct design alternatives* that can all achieve the desired performance. Because Target 3 is 2-dimensional, we present the data points that fit the rules in both the training and testing datasets in Fig. 3e. The result shows that the extracted data points can trace the design boundary nicely and fill the design boundary with reasonable coverage.

The results from this section demonstrate the capability for the tree method to design functional origami systems where multiple objectives with multi-physical performance indices are of interest. Moreover, the presented examples show that this methodology can provide alternative design options when they are available.

### Design for non-geometrical properties together with origami shape fitting

Finally, we demonstrate how the proposed method can enable origami shape fitting algorithms to further consider non-geometrical properties of the origami so that a holistic design can be accomplished. So far, most research on origami inverse design focuses on geometric shape fitting (such as those in^14,15,17^). Usually, the shape fitting problem can be constructed as an optimization problem, where the error between the target geometry and the origami is minimized given certain constraints^14,15^. However, these existing shape fitting studies cannot consider the non-geometrical properties that determine the functional performance of origami systems. Moreover, these shape fitting algorithms often leave tremendous flexibility for a designer to vary the origami pattern (e.g. number of panels used or maximum size of panels), without showing which combination may be better. Thus, this section will demonstrate how the proposed method can enable existing origami shape fitting algorithms to consider the interaction between the shape fitting and non-geometrical behaviors of the origami systems.

**Figure 5:**
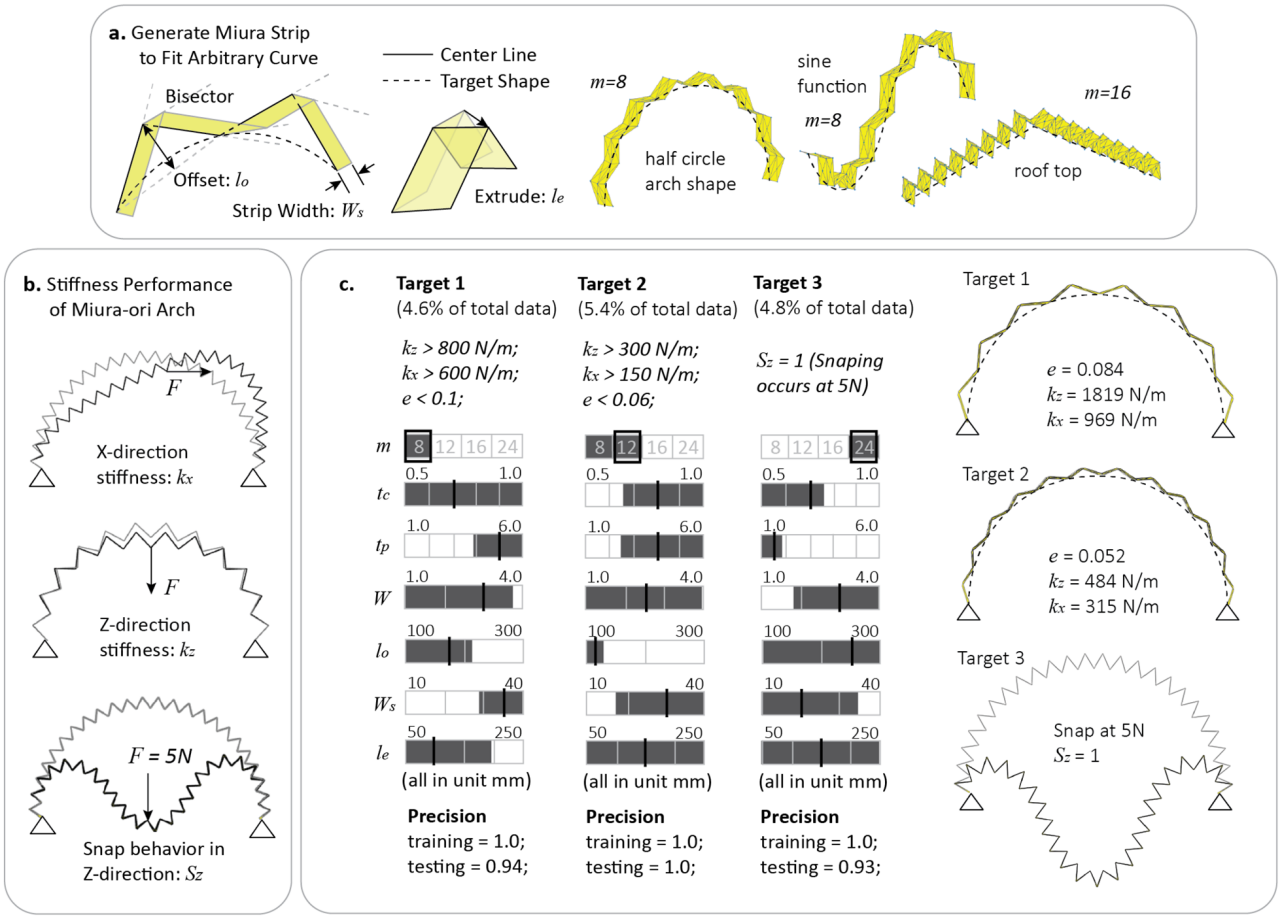
Integrating the machine learning method with shape fitting for holistic inverse design of origami. (**a**) Definitions for a modified Miura-ori design that can fit arbitrary curved shapes. Geometrical design features for the modified Miura include the number of units $m$, the offset length ${l}_{o}$, the width of the strip ${W}_{s}$, and the extrusion length ${l}_{e}$. (**b**) Four performance indices are studied including the error of fitting $e$, the stiffness in X and Z directions ${k}_{x}$ and ${k}_{z}$, and a binary variable ${S}_{z}$ that indicates if the structure will snap if loaded with a 5 N load in the Z direction. (**c**) Computed rules for three different targets and corresponding sample designs.

As a demonstration, we implement our method on top of an existing shape fitting approach introduced in^14^, where an analytical solution was derived to build Miura-origami strips to fit arbitrary planar curves. Figure 5a shows how this shape fitting method can generate different origami strips to fit a target planar curve. In this method, the target curve is first separated into a specified number of segments defined by $m$. Then a planar origami strip geometry is generated by setting the offset length ${l}_{o}$ of the center node and the width of the strip ${W}_{s}$. Finally, the 3D origami is created by extruding the planar geometry to form the Miura geometry with an extrusion length ${l}_{e}$. Figure 5a shows the shape fitting results for three different curves. As can be seen, there is great flexibility in selecting these parameters for shape fitting and the selection can now depend on which combination gives a more desirable non-geometric performance. Suppose our target is to build an origami structure that can achieve a given stiffness performance while fitting a target shape, how should we select these shape fitting parameters and other design features of the origami? The proposed machine learning based method is able to answer questions like this.

Without loss of generality, we focus on designing a Miura-origami half-circle arch with a 2 m radius. The origami arch database is generated by randomly picking shape fitting design features and other origami design features. The shape fitting features include the number of segments ($m=\{8, 12, 16, 24\}$), the offset length ($100 \, {\text{mm}}<{l}_{o}<300 \, {\text{mm}}$), the strip width ($10 \, {\text{mm}}<{W}_{s}<40 \, {\text{mm}}$), and the extrude dimension ($50 \, {\text{mm}}<{l}_{e}<250 \, {\text{mm}}$). Other design features such as the thicknesses of panels ($1.0 \, {\text{mm}}<{t}_{p}<6.0 \, {\text{mm}}$) and creases ($0.5 \, {\text{mm}}<{t}_{c}<1.0 \, {\text{mm}}$) and the width of creases ($1.0 \, {\text{mm}}<W<4.0 \, {\text{mm}}$) are also included because they affect the stiffness of the arch. Based on these features, we compute and record the responses of 3000 random origami designs using the SWOMPS simulation package to populate a database. The performance indices include the stiffness in X-direction (${k}_{x}$) and Z-direction (${k}_{z}$), the error of shape fitting ($e$), and whether the structure will snap or buckle under a 5 N load applied vertically (${S}_{z}$) (see Supplementary Materials Section S2 for details).

With the database established, we apply the proposed inverse design method to analyze the database. Because the decision tree-random forest method can handle a mixture of categorical and continuous variables, it can consider the integer variables used in shape fitting algorithm. Moreover, because the method can also tackle multi-objective problems, we can design for different combinations of shape-fitting errors and stiffness targets. Target 1 in Fig. 5c represents a target with stricter stiffness requirement but a less strict shape fitting objective while Target 2 has a more relaxed stiffness requirement but a stricter error objective. Both targets contain about 5% of the total data, so they are comparable in terms of overall design selectiveness. Figure 5c shows the computed decision rules for the two targets and indicates that both rules have reasonably high precision. This result demonstrates that the machine learning method can produce different design rules to accommodate the interactions between desired shape fitting error and mechanical performance. More importantly, the proposed machine learning method is not tied to specific origami patterns or shape fitting methods. Thus, the proposed methodology can be combined with other origami shape fitting approaches, such as those in^14,15,17^, to enable a holistic inverse design of origami that considers both shape and non-geometrical function or performance.

Next, we show that the proposed method can design origami systems with complex mechanical behaviors such as bistability and multi-stability^39,40^. Target 3 in Fig. 5c shows the design rules for an origami arch to exhibit a snap-through behavior under a vertically applied 5 N load. Unlike designing for a stiff arch with small fitting errors, the machine learning method shows that only the designs with more segments (larger $m$) in the strip can experience the snapping behavior. Moreover, it is necessary to have a low panel thickness and a low crease thickness so that the origami is more likely to snap. The testing precision of this design rule is high, indicating that the design rule is reliable and accurate.

Here, we have demonstrated one design scenario for shape fitting a Miura origami pattern to an arch geometry. However, we believe the versatility of the proposed framework can be readily extended to other origami shape fitting methods and even to freeform-origami design methods (such as those in^41^). For example, suppose that the origami geometry is allowed to slightly deviate from the target center line in order to achieve better mechanical behaviors. Then, in addition to the design features shown in Fig. 5, new xyz directional offsets of selected control vertices on the Miura arch can be used to generate different free-form variations of the geometry. With the directional offsets used as additional design features, it would be possible to simultaneously design for both the geometry and mechanical/non-mechanical performances of free-form origami structures.

## Discussion

In this section, we further discuss our reasoning on the implication behind the obtained results. We will start with data generation. In general, we believe reduced order simulation methods like the SWOMPS package^34^ are good for populating origami performance databases, because these methods can capture the multi-physical active folding in origami with reasonable accuracy^35,36^ and they are computationally efficient. To further improve the validity of the inverse design results, the populated origami performance database can be augmented with experimental data and/or high-fidelity FE simulations. By training the decision tree-random forest method with higher weight added to the experimental data points, the inverse design results can be made more representative and realistic.

Next, when labeling the dataset based on target performance, we find that it is common to generate imbalanced databases because those data points that meet our target performance tend to be sparse (engineering design usually targets rarer performances). It is possible to make the database more balanced by using domain knowledge to preselect sample zones or even rule out non-relevant design features. Nonetheless, this work shows that the proposed method can handle the inverse design problem without using techniques to balance out imbalance databases. This work uses the sklearn package to train the decision tree-random forest method and uses the embedded imbalanced weight to handle the imbalance dataset.

This work demonstrates that it is possible to find the representative combination of design features for inverse design of origami systems by finding the tree branches with higher F-score. This strategy is similar to finding the better classifier from a pool of classifiers using the F-score, but focusing on a sparser tree branch. Focusing on a few high performing branches in the decision tree-random forest method produces design rules with high interpretability for solving the inverse design problem. Analysis from Section S3.4 shows that better inverse design results can be obtained by using larger origami dataset and the method is not affected by overfitting issues because using a tree branch for inverse design enforces a highly sparse structure. Moreover, results in Section S3.5 further highlight that the proposed methodology can produce stable inverse design results under different training/testing data partition, which is usually not achievable when using a single decision tree. These results show the benefit of focusing on sparser tree branches for solving the inverse design problem than using the standard decision tree method.

In summary, this work establishes a novel inverse design method for functional origami structures using an interpretable machine learning method. First, the origami performance database can be populated using reduced-order simulation methods like the SWOMPS package. After populating the databases, the data points are classified into two classes depending on whether they meet the specified target performance, which casts a binary classification problem. After setting up the target performance, the decision tree-random forest method was trained to identify origami patterns and design features in origami structures that achieve a specified target performance. Finally, origami design rules were computed by backtracking the splitting criteria associated with each tree branch and selecting the rules with the highest F-score.

To test the performance and versatility of the proposed method we built databases for four design scenarios, including: (1) Stiffness of a single unit Miura-ori, (2) Stiffness of origami metasheets, (3) Multi-physical performance of electro-thermal origami grippers, and (4) Stiffness and shape fitting of origami arches. We believe these four databases populated in this work can be reused for generating new designs or for testing the performance of other machine learning algorithms when applied to analyze origami related data.

Future research could explore and improve the reliability and performance of the proposed method by using other state-of-the-art training methods for decision trees (like the Generalized and Scalable Optimal Sparse Decision Trees^42^ and the Optimal Classification Trees^43^). In addition, it would be possible to make the origami performance database more representative by including data points generated with high fidelity FE simulations and/or experiments. Addressing potential shifts between the distribution of simulated and experimental data and assigning different weights to the simulated and experimental results are two interesting problems that would need further investigation. Moreover, the inverse design results presented in this work have not yet been experimentally verified and tested. Physical experiments could be used to judge the performance of this inverse design framework under realistic engineering conditions.

As a concluding remark, we want to summarize the benefits of using this data science and interpretable machine learning based approach for origami inverse design when compared to using existing optimization-based strategies. First, any generated database can be reused to compute new rules for different targets, or in other words to find appropriate designs for different scenarios. Second, the proposed method can simultaneously analyze the significance of design features for a given design target, which is not provided in optimization-based design methods. If a design feature is important for achieving a given performance target, a relatively tight threshold of that feature will be identified. Third, the proposed method demonstrates the complex interaction between continuous variables and categorical variables. Identifying these interactions is necessary for designs where comparing different origami patterns will naturally introduce categorical variables that cannot be captured with continuous optimization-based design methods. Fourth, we show that the proposed method can handle multi-objective design targets for active origami systems with multi-physical behaviors. Finally, we demonstrate that the proposed method can extend existing origami shape fitting algorithms to further design for non-geometrical performance of origami structures, which together enables a holistic framework for inverse design of functional origami. We envision that the proposed methodology can be used for designing active origami systems with superior performance for various applications in biomedical devices, soft robotics, metamaterials, deployable structures, and many more.

## Methods

### Origami simulation

This work uses an open-access origami simulation package called SWOMPS^34^. This origami simulator uses a common simulation technique called the bar and hinge model to represent the geometry of origami systems. This simulator can explicitly model compliant origami creases (folds with distributed width) which makes it suitable for simulating the behaviors of practical origami structures^35^. In addition, the simulator package integrates a state-of-the-art multi-physics model to capture the electro-thermal actuation^36^ important for active origami assemblages. Other origami simulation techniques may be used for capturing different performance of interest, and the reference^44^ provides a summary of different origami simulation techniques. The implementation codes for building origami databases are thoroughly discussed in the Supplementary Materials and are available on the GitHub page: https://github.com/zzhuyii/GenerateOrigamiDataSet. A more detailed introduction of the underlying origami simulation method and how it is applied in each design example can be found in the Supplementary Text Sections S1 and S2.

### Training the decision tree-random forest method

This work uses an open-access package sklearn^36^ to train the decision tree-random forest machine learning method. More specifically, the sklearn package implements a classical decision tree method, where the trees are trained using suboptimal greedy approaches. Other state-of-the-art tree methods (like the Generalized and Scalable Optimal Sparse Decision Trees^41^ and the Optimal Classification Trees^42^) could be used in the future to further improve the performance. When training decision trees, an entropy-based criterion is used to identify the best splitting rules at branch nodes. Because the target class tends to contain only a small number of data (5% to 10%), the balanced class weight is used to tackle the imbalanced dataset. The results computed in the main article are accomplished using the following hyperparameters: the cost-complexity-pruning alpha value is 0.001, the maximum depth of trees is 20, and the number of training trees is 100. The details on the hold-out testing and hyperparameter selection can be found in the Supplementary Text Section S3.

### Finding the inverse design rules

This work shows that each tree branch for data points that meet the target performance can be treated as a design rule for an origami system. With this in mind, the work further shows that it is possible to find the more representative design rules (tree branches) using the F-score function. To achieve this inverse design method, we developed an open-access code package to analyze the trained random forest classification model, and to back track all tree branches (design rules) associated with the class that meet the target performance. This new code implementation then ranks the design rules and picks the most representative one using the proposed selection methodology. Further details on the inverse design workflow can be found in the Supplementary Document Section S3. The implementation code package of this work can be found on GitHub: https://github.com/zzhuyii/TreeForOrigami.

### Evaluation criterion

Common evaluation indices for machine learning, including the precision and the recall, are used to evaluate the performance of design rules. The precision is defined as the ratio between the number of accurate predictions of Class *t* over the number of all predictions of Class $t$^38^. In Fig. 2e, Rule 1 (blue dots) predicts 10 data points as Class 1, and 9 of them are correct so it has a precision of 0.9. The recall is defined as the accurate predictions of Class $t$ over the number of all data of Class $t$^38^. In Fig. 2e, suppose we have a total number of 30 points in the target zone (within the blue decision boundary), then Rule 1 will have a recall of 0.3 (9/30).

## Supplementary Information

Supplementary Information 1.

Supplementary Information 2.

## Data Availability

All data and code used for the analyses are available in the main text or the supplementary materials.
