# Supplementary material for: Harnessing interpretable machine learning for holistic inverse design of origami
Source: Sci Rep. 2022 Nov 11;12:19277. doi: 10.1038/s41598-022-23875-6 (PMC9652322; doi:10.1038/s41598-022-23875-6)
Supplement: Supplementary file 1 — Supplementary Information 1. [file 41598_2022_23875_MOESM1_ESM.pdf]

Supplementary Materials for

Harnessing Interpretable Machine learning for Holistic Inverse Design of Origami

Yi Zhu*, Evgueni T. Filipov

*Corresponding author. Email: yizhucee@umich.edu

This PDF file includes:

Supplementary Text
Figs. S1 to S10
Table S1
References (1 to 12)

Other Supplementary Materials for this manuscript include the following:

Data S1 to S4
Codes S1 to S2

Supplementary Text

S1. Introduction of Bar and Hinge Models

This section describes the bar and hinge model used to generate the origami performance database for the manuscript. The bar and hinge model is a widely used reduced-order model for simulating the mechanical behaviors of origami systems [1, 2], and it contains two major elements: the bar element and the rotational spring (hinge) element. The bar elements are extensional 3D springs that can capture in-plane behaviors of origami such as the stretching and shearing deformations (see Fig. S1b). The rotational spring elements are rotating hinges with stiffness that can capture the out-of-plane behaviors of origami including the panel bending and crease folding deformations (see Fig. S1b).

More specifically, this work uses the origami simulation package SWOMPS published in [3] where the bar and hinge model is enhanced to capture the multi-physical behaviors important for active origami systems. The SWOMPS package implements both the standard bar and hinge formulation where the folding creases are simplified as 1D rotational hinges and the compliant crease bar and hinge formulation where the compliant crease regions are captured with distributed widths [4] (see Fig. S1a). The package also supports the simulation of multi-physics-based electro-thermal crease actuation [5].

A potential-based static simulation is implemented in this simulation package. The nonlinear folding behavior of active origami systems can be traced using different nonlinear PDE solvers. In general, the internal potential of the active origami can be expressed as:

$$U_{ori}(\boldsymbol{x}, Q) = U_{bar}(\boldsymbol{x}) + U_{spr}(\boldsymbol{x}, Q),$$

where $\boldsymbol{x}$ represents the nodal coordinates of the origami system and the terms U_{bar}, U_{spr} are the potential energies associated with the bar stretching and spring rotation respectively. The applied heating power Q can affect the stress-free configurations of the rotational springs and thus, change the potential of springs. Linear elastic bar elements and rotational spring elements are used in this work so the potential of bar element is:

$$U_{bar} = \frac{1EA}{2L_0}(L(\boldsymbol{x}) - L_0)^2,$$

where L is the bar length, L_0 is the original length, E is the Young's modulus, and A is the area of the bar element. Similarly, the potential of the rotational spring is:

$$U_{spr} = \frac{1}{2}k_{spr}\big(\theta(\boldsymbol{x}) - \theta_0(Q)\big)^2,$$

where k_{spr} is the spring stiffness, θ is the rotational angle, and θ_0 is the stress-free rotational angle which is a function of the applied heating Q. Detailed derivations of the stiffness parameters (e.g. A, k_{spr}) can be found in [1, 4].

Finally, Fig. S1c shows how the bar and hinge model can capture heat transfer within electro-thermally actuated origami structures. Because active origami structures tend to be small and

operate at a low temperature, there is limited convection and radiation within the system. Therefore, we can capture the heat transfer within the origami system and heat loss from the origami structure to the ambient environment using simplified conduction models. The heat transfer within origami is captured using linear triangular thermal conduction elements and the heat transfer between the origami and the surrounding environment is simulated as a simplified 1D heat conduction problem (see Fig. S1c). Reference [5] provides further details on the formulation, verification, and validation of this simplified simulation method.

Solving for the equilibrium of the origami under applied loads or applied heating power Q can be achieved by finding the local minimum (extremum) of the total potential energy:

$$\frac{\partial U_{ori}(Q)}{\partial \boldsymbol{x}} = F_{ext}.$$

A number of nonlinear PDE solution methods can be used to find the equilibrium configuration associated with a given loading or applied heating [5, 6]. The SWOMPS package provides the Newton-Raphson Method, Displacement Control Method, and the Modified Generalized Displacement Control Method for solving the equilibrium. In this work, one of these three methods is used to solve for the equilibrium of the origami depending on the type of the problem.

Using the SWOMPS package for generating the performance database is beneficial because of the following reasons. First, this package can capture the electro-thermally actuated folding creases so that we can study the power consumption and maximum temperature of active origami structures. Second, the SWOMPS package is computationally efficient, so it is possible to populate an origami performance database rapidly. Finally, this package is verified against experiments, so the populated database is representative of realistic origami behaviors. The bar and hinge origami simulation codes and the computed origami performance databases can be found on GitHub or in the supplementary material of this manuscript.

S2. Generating Origami Performance Database

This supplementary section describes how we populate the three more complex origami performance databases demonstrated in the main text. The execution codes for all three examples are published as supplementary materials and can be found on GitHub. Links to the GitHub website are provided in the final section of this supplementary text.

Figure S2 shows how we built the origami canopy database. Two different origami patterns are used and they are represented using a categorical feature $p = \{1,2\}$, meaning patterns 1 and 2. We also consider the number of cells in the x and y directions ($m = \{24,30,36\}$ and $n = \{6,9,12\}$, respectively) as categorical design features. Other design features are continuous and include the thickness of panels (t_p) and creases (t_c) and the width of the creases (W). We assume the material used to fabricate the origami has a Young's modulus of 2 GPa, which represents common polymeric materials. Furthermore, we use a standard bar and hinge model where the crease folding stiffness is represented using a single rotational spring. This spring stiffness value is calculated using a pseudo-rigid-body model (see Fig. S2c). The calculation of other stiffness parameters follows the method introduced in [4]. When solving for the global stiffness of the canopy structure, the left end of the origami are fixed in space (no translation in xyz directions) while the right end

are loaded with uniformly distributed forces. We apply a total of 3 N force and use a Newton-Raphson loading scheme to compute the resulting displacement. The global stiffness is calculated as the ratio between the applied load and the nodal deformation. Two thousand (2000) samples with randomly generated feature values are calculated for each pattern, and the database contains both the bending and axial stiffnesses at 30%, 60%, and 90% extension. The extension is measured as the ratio between the folded length of the pattern L' and the original length L where the origami is a flat and unfolded sheet.

Figure S3 shows how we build the database for active origami grippers. Figure S3a shows the geometrical definitions of the three origami patterns used to build the grippers, where the red solid lines are valley folds and the blue dotted lines are mountain folds. A categorical feature $p = \{1,2,3\}$ is used to represent these three different patterns. Other design features are continuous and include the length (L_1) and width (L_2) of the gripper arm, the location of the first hinge from the base (measured as a ratio Ra of the outer arm compared to the total arm length), the thickness of the two layers in the actuator design (t_1 and t_2), and the width of the actuator creases (W). All creases in these patterns are assumed to be bi-layer electro-thermal actuators similar to those in [7]. We assume that both the active layer and the passive layer of these actuator creases have a Young's modulus of $E_1 = E_2 = 2$ GPa and the difference between the thermal expansion coefficient is $\Delta\alpha = 50\times10^{-6}$. We also assume that the origami system has a density of 1200 kg/m^3, which is representative of common polymeric materials. The fundamental frequency of the system is computed using a particle bar and hinge formulation, where the mass of the structure is lumped at the nodes of the origami. In this example, a compliant crease bar and hinge formulation is used to represent the distributed geometry of the active creases, where the folding of an active crease is represented using three lines of rotational springs [4]. Electro-thermal heating is applied uniformly to all creases to trigger the actuation. We solve the applied heating power Q needed to close the gripping arm by incrementally increasing the applied power with a step of 0.1 W/m (applied heating per length of creases). This electro-thermal folding motion is captured using a simplified origami simulation model introduced in [5]. After the gripper is closed with the actuator creases, a Newton-Raphson loading scheme is used to study the stiffness of the gripping arm. A small force (1×10^{-8}N) is applied outward at the tip to calculate the stiffness of the gripper for resisting forces prying it open. Two thousand (2000) sample grippers for each pattern are simulated to create the database.

Finally, we present the details on how we build the origami arch database (Fig. S4). To fit an origami strip to an arbitrary curve, the target curve is first separated into different segments determined by the number of segments $m = \{8,12,16,24\}$. Then, the centerline of the Miura origami geometry is determined using the offset distance l_o, where the center node is moved towards one direction of the curve from the center point by l_o. After determining the centerline, the planar Miura geometry is determined using the bisector angles and the width of the strip W_s. Finally, the 3D Miura origami is generated by extruding the 2D planar geometry using the length l_e. Further details on this shape fitting method can be found in [8]. In this origami arch database, the target curve is a half-circle with a radius of 2m. After the Miura half-circle arch is generated, its mechanical performance can be calculated using the SWOMPS simulation package. The support of the arch is set by fixing the three nodes at each end of the strip in 3D space. We assume the material has a Young's modulus of 2 GPa and a Poisson's ratio of 0.3. A concentrated force of 5N is applied at the middle of the arch as shown in Fig. S4b. The deformed configuration is solved using a Newtown-Raphson solver, and the stiffness is calculated as the ratio between the applied load and

the nodal displacement (as secant stiffness). The snapping behavior is determined by judging if value of the nodal displacement has exceeded 1m. Three thousand (3000) samples are calculated to build this database.

S3. Compute Interpretable Design Rules with Decision Tree-Random Forest Method

In this supplementary section, we will introduce the decision tree-random forest method, which is an inherently interpretable machine learning method. First, we give a brief introduction on the formulation and the training process of the tree method. Next, we will talk about the hyperparameter selection and introduce one technique called one-hot-encoder to integrate categorical features into the sklearn implementation package for tree methods.

S3.1 Introduction to Decision Trees and Random Forests

Figure S5a shows how a decision tree classifier works. There are two types of nodes within a decision tree and they are branch nodes and leaf nodes. Consider a classification problem where the decision tree is used to differentiate two classes. To predict the class of a data point, the data point can be sent into the decision tree from its root branch node (the branch node at the top). The data point will then either flow to the left or to the right depending on whether it meets the criterion associated with the branch node or not. Finally, after a number of judgements, the data point will arrive at a leaf node where it will be predicted to be either class 1 or class 2 (for this binary classification problem). As shown in Fig S5a, the formulation of a decision tree is highly interpretable and easily understood by humans. This is one major advantage of using interpretable machine learning methods over traditional "black-box" machine learning methods like neural networks [9].

Figure S5b shows how a decision tree classifier can be trained. The classical method for building decision trees is a sub-optimal greedy method. Instead of building the entire tree all-at-once with optimized performance, the tree is built in a node-by-node manner. Suppose we start with a training data set, which is a mixture of data classified either as class 1 or 2. Then, the decision rule of the root node is designed such that after the separation, the two new datasets are "more pure" than the original combined input dataset. The purity of the dataset can be measured using the entropy defined as:

$$\sum_{c=1}^{C} -\frac{n_c}{n} log\frac{n_c}{n},$$

where c is the class of data, n is the number of data, and n_c is the number of class c data. For a two-class classification problem, we have $c = \{1,2\}$. Based on this definition, the purer the data, the lower the entropy value will be. Other similar criteria such as the Gini index can also be used in addition to the entropy. When selecting the decision rule of a branch node, different potential rules are enumerated to find the one that can produce the lowest entropy after the separation.

After producing the two new sub datasets, we can then determine if we should keep splitting the new dataset or not (creating a new branch node). If the sub datasets are still not pure enough and the tree has not yet reached the specified maximum depth, the algorithm will redo the process and obtain a new splitting rule. However, if the sub datasets are pure or if the tree has reached the

maximum depth, the algorithm will stop and generate the leaf nodes. The prediction label of a leaf node is determined by the most commonly occurring class of the training data separated into that leaf node.

Because the decision trees are built node-by-node, we can only obtain sub-optimal trees with this approach. There are also methods to create optimal decision trees such as the OCT method demonstrated in [10], but this formulation requires solving mixed-integer-programs, which is much more time consuming than using a greedy approach. Therefore, this work uses the standard greedy approach to generate the decision tree.

In practice, it is more common to use the ensembled version of the decision tree method, the random forest method, for better performance. The formulation of a random forest is straightforward, and it simply means generating different decision trees and taking the most common class prediction result out of all trees. One way of obtaining different decision trees is to use random subsets of the training data for the training process (see Fig. S5d). This work uses the random forest method to create drastically different tree branches for better inverse design rules.

S3.2 Techniques for Integrating Categorical Features

In this work, the decision tree method is implemented using the widely used sklearn package [11]. Although the decision tree method can consider categorical data theoretically [12], conventional implementations of the decision tree (such as the one in the sklearn package) may not directly support the use of categorical data. In the default setting of the sklearn package, all variables are treated as continuous numerical values. However, there is a technique called one-hot encoder that can be used to resolve this problem.

Considering that we have a categorical feature called "type of origami pattern" p and the values for this feature include $\{1,2,3\}$. Without using the one-hot encoder, the sklearn package will treat m as a continuous numerical variable, which means that the machine learning algorithm can potentially create a rule in the form of "pattern $p > 0.5$". This is inaccurate because $p = 2$ and $p = 3$ are categorical features that cannot occur at the same time. To fix this issue, we can use the one-hot encoder to convert the integer input data (see Fig. S5c). The categorical variable p ($p = \{1,2,3\}$) is converted to three different binary variables as "$p = 1$", "$p = 2$", and "$p = 3$", where each data point can take values of 1 for true or 0 for false. With this formulation, the sklearn package can treat the variable p as categorical through using the encoded binary true/false variables. Using the one-hot encoder introduces a large number of sparse input features that can potentially make the algorithm computationally inefficient. In such scenarios, it would be more effective to select a dedicated implementation package of the tree method that can directly support categorical features.

S3.3 Hold Out Test and Hyperparameter Selection

Here, we introduce the hold out testing setup and hyperparameter selection process that are needed to use the decision tree method. Figure S5d gives an illustration of the hold out testing setup. The idea of doing a hold out testing is to check the performance of our trained machine learning algorithm. What we do is to 'hold out' a number of data from the full database while training the machine learning algorithms and only use these data when we are testing the performance of the

machine learning. It is similar to creating two sets of questions: one set for training the students, and one set for testing how well the students learned from working out the questions in the training set. In this work, the complete dataset is randomly split into two sets: one with 60% of the data for training the machine learning method, and the other one with 40% of data for testing the performance.

Hyperparameters refer to the variables in the machine learning algorithm that are explicitly specified by the users and are not learned from the training data. Usually, a grid search is performed to find the optimal hyperparameter values for the machine learning algorithm. In this work, we consider five hyperparameter variables and they are (1) the maximum depth of the trees, (2) the number of tree learners in the forest, (3) the cost-complexity pruning alpha value. (4) the splitting criterion, and (5) the subset ratio for training. The precision of the selected final design rule is used to assess the choice of each hyperparameter. Five runs of different hold out tests are used, and the mean precision is recorded. Because the depths of trees and the number of tree trainers are the two major hyperparameters of a decision tree-random forest method, we mainly focus on the effects of these two values while only briefly highlighting the others. In addition to the grid search results, we also trained two other machine learning algorithms for comparison, namely the kNN method, and Gradient Boosting method. The kNN method is usually used as a baseline method in machine learning because it has a simple formulation. In general, we expect the precision obtained from the target method be superior to that obtained using the kNN method. The Gradient Boosting method is a “black box” machine learning method that tends to perform well on many datasets. If the target method can achieve a precision that is close to that from the Gradient Boosting, the target method can be seen as competitive. Table S1 shows a comparison between the prediction precision of the decision tree-random forest method, the kNN method, and the gradient boosting method. The results are obtained for the 8 design targets shown in Fig. 3 using the origami metasurface database. We want to emphasize here that the results in this table are only for class prediction precision and are not the inverse design precision. Because standard machine learning methods like kNN and gradient boosting cannot do inverse design, it is not possible to compare the precision of the entire inverse design process.

We then perform three rounds of grid search for the origami canopy (Fig. S6), the origami gripper (Fig. S7), and the shape fitting origami arch (Fig. S8) databases. For the hyperparameter selection, we are using the inverse design precision as the selection criterion not the class prediction precision. First, we give a brief discussion on the influence of the maximum depth of trees and the number of trees in the forest. We can see from Fig. S6 to S8 that there is a saturation effect in the maximum depth of trees. The precision improves when increasing the maximum depth but stops once the maximum depth reaches certain values. This saturation is expected because if tree-based learners are too deep, the leaf nodes will just have one or two data points, which is seen as “pure” by the algorithm. In this case, further increasing the depth of trees will not change the tree structure. Moreover, branches like these are not representative and cannot be used to compute reliable design rules. Therefore, it is recommended to set the depth of trees so that we barely reach that saturation point. In this work, we select a depth of 20. For the number of tree trainers in the forest, we found that a relatively large number of tree trainer is helpful for improving the precision. However, when the value is too large, the precision can also saturate. This saturation happens because we may be close to “enumerating all possible” tree branch formulations when using a large number of tree trainers. In this case, adding tree trainers may not generate new branches we want. Thus, a value of 100 is picked for the number of trainers in this work.

Figure S6 studies the effects of three hyper parameters: cost-complexity-pruning alpha (ccp-alpha) value, depth of trees, and number of trees. With the grid search result for the origami canopy dataset, we further study the effects of the cost-complexity-pruning alpha (ccp-alpha) value. The cost-complexity-pruning is another step in training decision tree methods where the machine learning method will automatically remove some branch nodes from a tree to avoid over-fitting. In general, the larger the alpha value is, the more likely the algorithm will remove nodes. As demonstrated in Fig. S6, the pruning alpha value does not have a significant influence on the precision of our prediction, and increasing the alpha value does not improve the precision for all cases. Therefore, this work uses a ccp-alpha value of 0.0001 in all the training performed.

Next, we want to study the effects of splitting criteria with a grid search on the origami gripper database. Thus, Fig. S7 studies the effects of another three hyper parameters: splitting criterion, depth of trees, and number of trees. The sklearn package provides two different splitting criteria and they are the Gini index and the entropy index. The Gini index is similar to the entropy but is computed differently [11]. From Fig. S7 we see that entropy tends to provide better performance than the Gini index. Therefore, we use entropy as the splitting criterion in our work.

Finally, with the origami arch database, we further consider the effects of the subset ratio. Thus, Fig. S8 studies the combined effects of three hyper parameters: subset ration, depth of trees, and number of trees. To train an effective random forest algorithm, different decision trees need to be built [12]. One way of achieving this goal is to use a randomly generated subset of the training data to train the decision trees instead of using the entire training database. The subset ratio is the ratio between the size of this sub dataset when compared to the entire training database. The results of the hyperparameter grid search are shown in Fig. S8. We can see that a smaller subset ratio can improve the performance of the machine learning algorithm, because a smaller subset ratio can encourage the algorithm to generate drastically different branches for computing the better decision rules. Thus, we select a subset ratio of 0.5 in this work. We did not explore the potential of having an even smaller subset ratio value because that will make the training set too small.

S3.4 Influence of Dataset Sizes and Computational Time

Here, we take a closer look at the influence of dataset sizes on the testing precision and the computational time. We use the origami metasurface database (without adding material property as design features) and the shape fitting database to study the behavior (see Figure S9). For the metasurface database, the target is to find an origami metasurface at 60% deployment with an axial stiffness above 80000 N/m. For the shape fitting dataset, the target is to find an origami with a stiffness in the z-direction greater than 800N/m, a stiffness in the x-direction greater than 6000N/m, and a shape fitting error smaller than 0.1.

As can be seen, the testing precision first improves and then saturates as the training data size increases. When a small dataset is used, the data is not enough to represent the complex interaction between the design features so there is an under fitting behavior reflected in the low testing precision. However, we do not observe an overfitting behavior with the training data size we have, and the testing precision is kept more or less constant when we have large training datasets. We believe the formulation of the method is less likely to experience over fitting issues because of the high sparsity in using tree branches for inverse design. Finally, we present the training time needed to train the decision tree-random forest and to find the representative combination of design

features. As can be seen, the proposed methods are computationally efficient and the entire training and inverse design process can be done in 10 seconds for the types of datasets we are using. The relatively high efficiency allows the method to be potentially scaled up for more complicated origami design problems with higher dimension design features.

S3.5 Reliable and Stable Inverse Design

In this section, we want to demonstrate that the proposed inverse design method can give reliable and stable inverse design result. One concern regarding using a decision tree method for solving engineering problem is that decision trees are “unstable”. That is to say, using different training/testing data partition will results in drastically different decision trees. Therefore, ensemble versions of trees, like random forest, are used to “average out” the variabilities to obtain better testing accuracy. Here, we want to demonstrate that focusing on a single branch of the decision tree for inverse design is actually much more stable than training a decision tree. To demonstrate this, we show the inverse design result for soft origami arch using different training/testing data partitions on the shape fitting database (see Fig. S10). The design target is to find an origami arch that will experience the snap through behavior and the results in Fig. S10 show two stable clusters of inverse design results when we use different training/testing partition. These clustering happens because of the complex interaction between categorical design features and continuous design features similar to the results obtained in Fig. 4d in the manuscript. Despite finding two both competitive clusters of inverse design result, the design rules within each clusters are highly similar to each other. This finding really highlight the stability of the proposed inverse design method and show that we can obtain stable inverse design rules from tree branches even if the entire tree structure can be unstable.

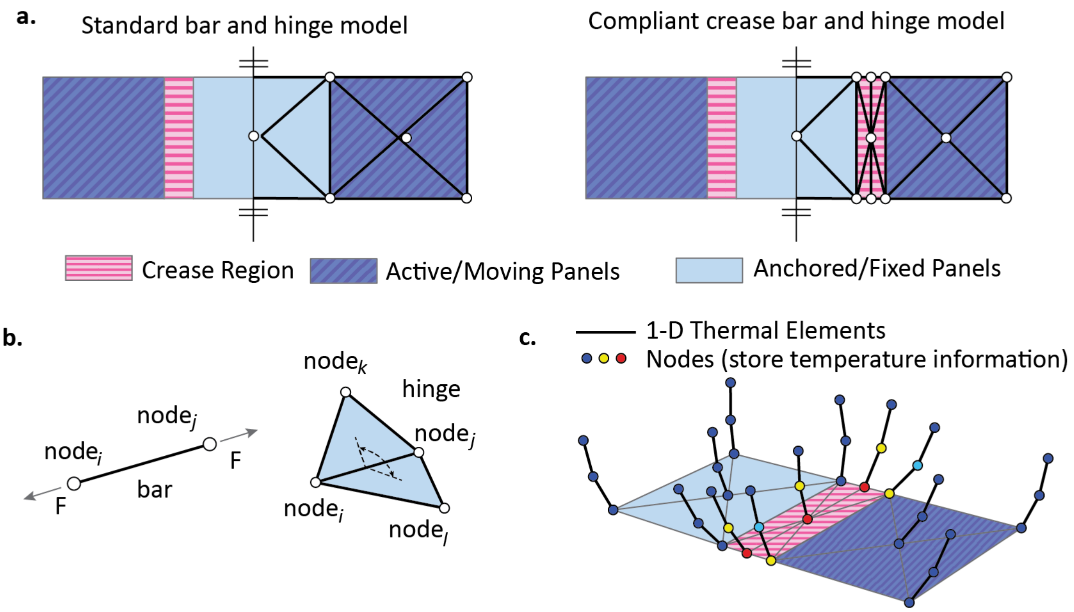

Fig. S1. Bar and hinge models for simulating multi-physical behaviors of origami systems. a. In this work, we use both the standard bar and hinge model where a folding crease is simplified to a 1D rotational element, and the compliant crease formulation that simulates the distributed crease region. **b.** The bar and hinge model uses bar elements to capture in-plane behaviors and rotational springs (or hinges) to capture the out-of-plane behaviors. **c.** A reduced order heat transfer model is used to capture the thermal distribution and heat-driven actuation of active origami systems.

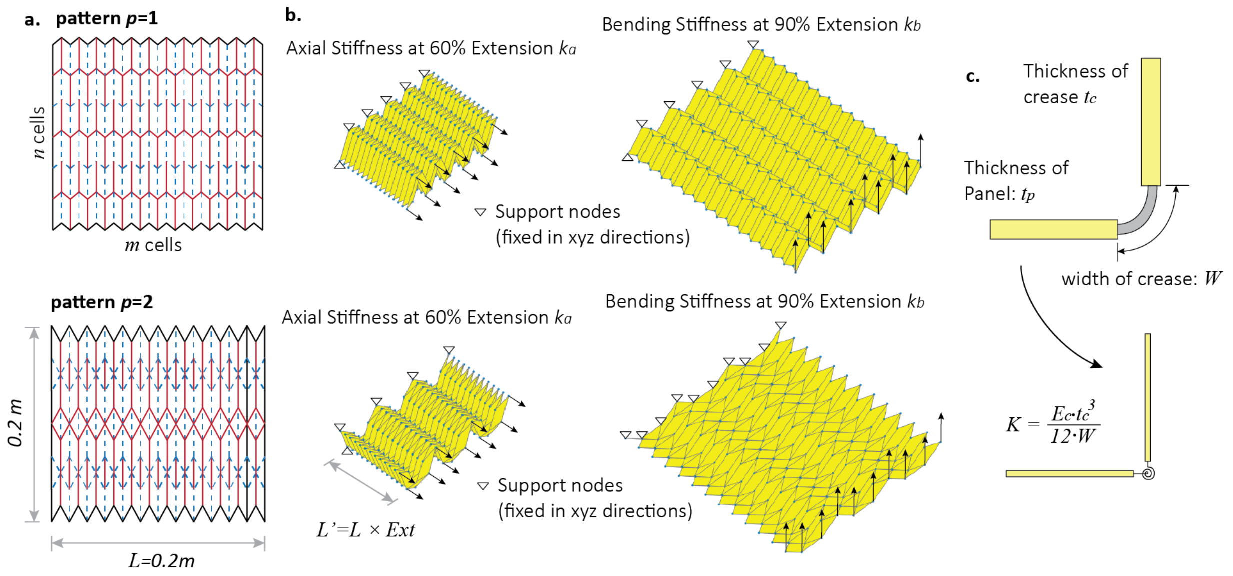

Fig. S2. Details on building the canopy database. a. The geometry of the Miura origami pattern (p=1) and the Tachi-Miura-Polyhedron (TMP) origami pattern (p=2). Both canopies are cut from 0.2m square sheets with the same thickness and material properties. **b.** The simulation setup for building the database. The nodes at the left end are fixed in 3D space while a load is applied on the right end. The global stiffness properties are calculated at different extension ratios (*Ext*) of the origami. **c.** A pseudo-rigid-body model is used to calculate the rotational spring stiffness of the creases in the origami canopies.

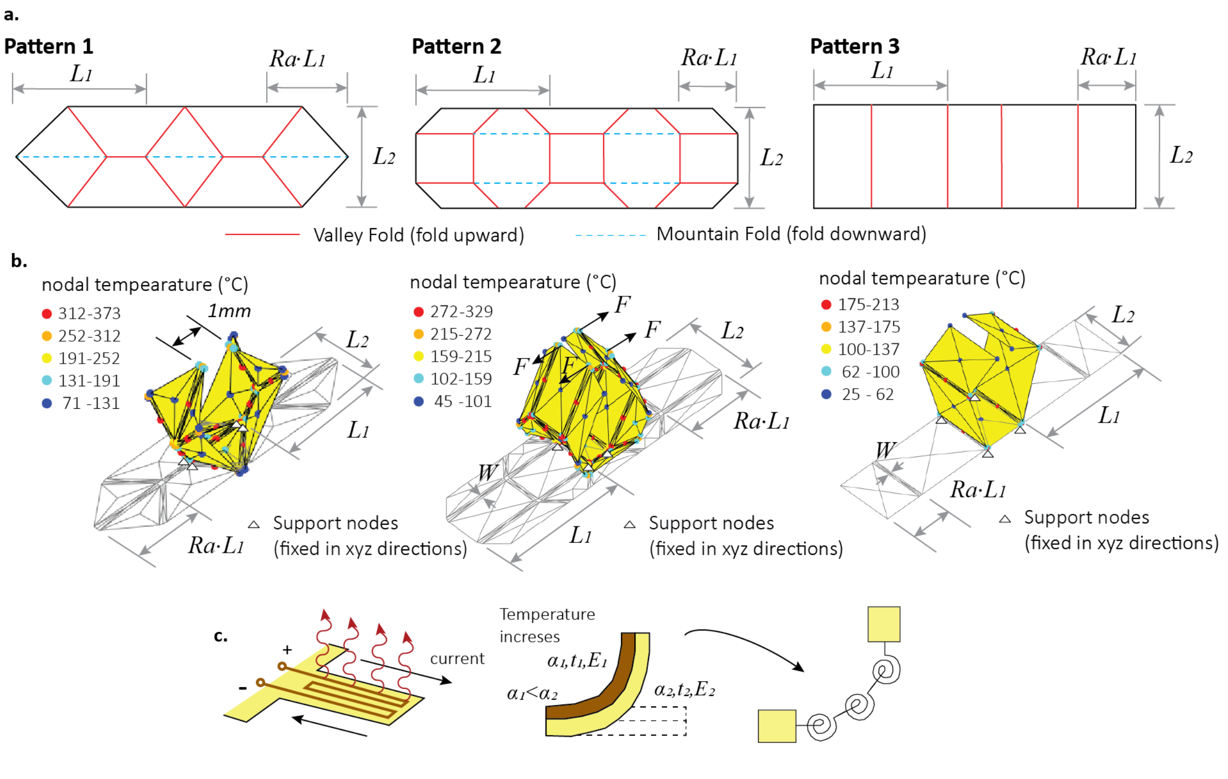

Fig. S3. Details on building the active origami gripper database. a. Geometrical definition of the three origami patterns used to build active grippers. **b.** Folding simulation of the three grippers. Four nodes at the base of each pattern are fixed in 3D space to serve as the supports. An electro-thermal loading is applied to close the gripper arms to be less than 1mm apart. Then, forces are applied onto the gripper tips to pry it open and the stiffness is recorded. **c.** A compliant crease bar and hinge model is used to model the electro-thermally driven bi-layer actuators at the active crease regions. The distributed curvature of these active creases is represented using three lines of rotational springs.

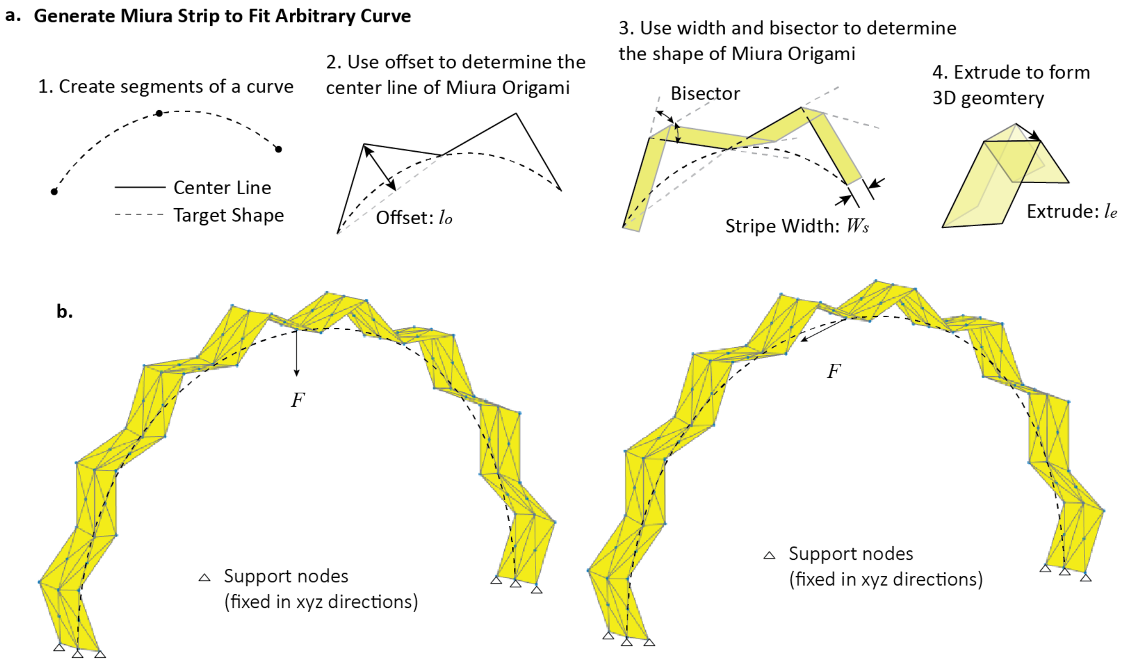

Fig. S4. Details on building the database for origami shape fitting. a. The four-step method to generate the Miura strip for shape fitting. **b.** Loading simulation of the origami arch. A concentrated force is applied at the center of the arch and the stiffness is calculated. The three nodes at each end of the arch are fixed in 3D space to serve as supports.

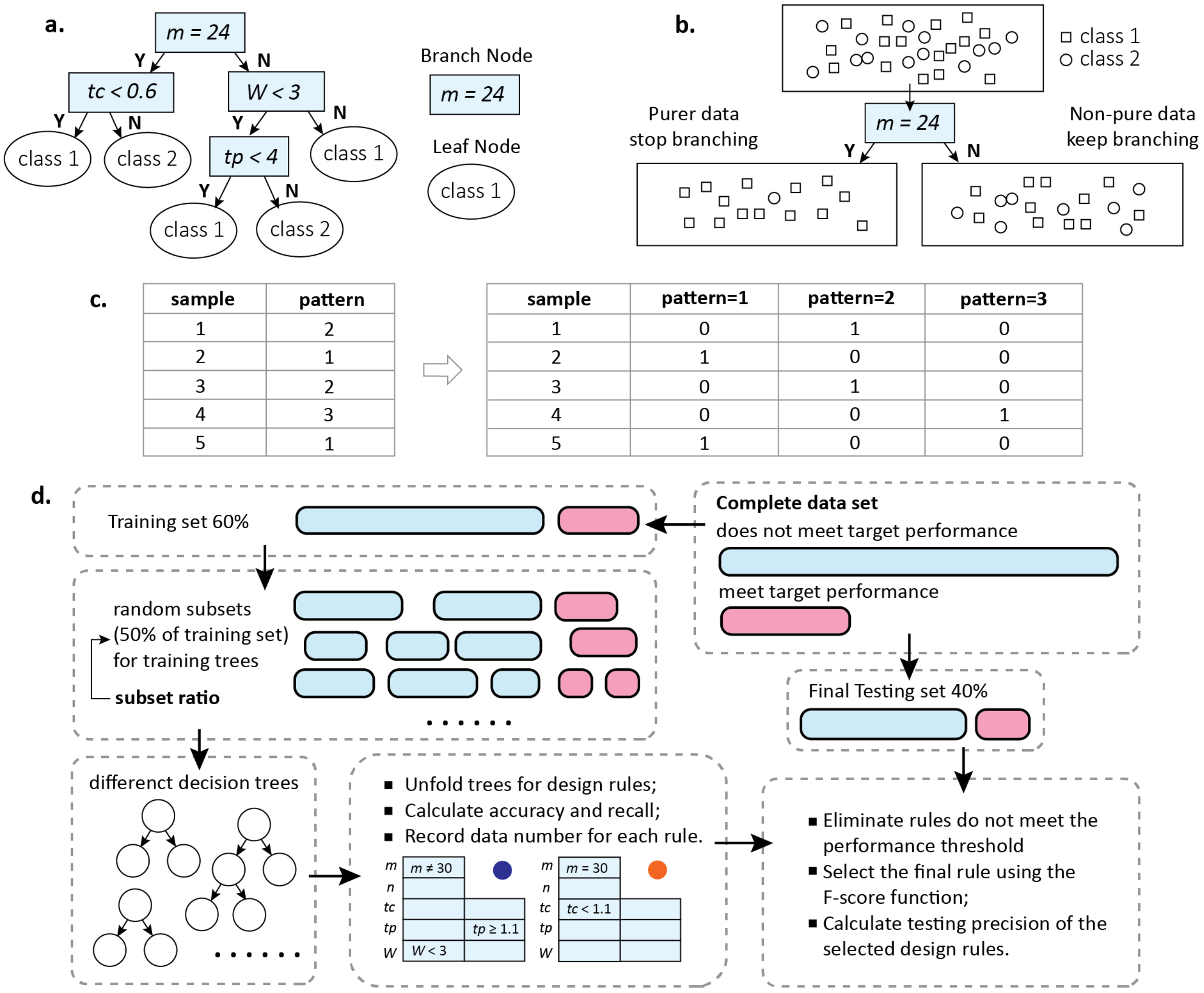

Fig. S5. The formulation of a decision tree method. a. A sample decision tree for a binary classification problem. **b.** The decision rule of a branch node is computed so that more pure sub data bins can be obtained after separating the data using the rule. **c.** Using a one-hot encoder allows the sklearn decision tree package to handle categorical data. **d.** Hold out test setup for checking the precision of rules.

Origami Metasurface (60% bending stiffness < 15000 N/m; 90% axial stiffness < 150000 N/m; 700 N/m < 90% bending stiffness < 1600 N/m;)

Precision of kNN: 0.639; Precision of Boosting: 0.748

ccp_alpha = 0.0001

tree number	20	40	60	100	200
depth=8	0.837	0.769	0.769	0.913	0.910
depth=12	0.848	0.826	0.929	0.933	0.911
depth=16	0.848	0.826	0.927	0.933	0.913
depth=20	0.848	0.826	0.927	0.933	0.913
depth=24	0.848	0.826	0.927	0.933	0.913
depth=28	0.848	0.826	0.927	0.933	0.913
depth=32	0.848	0.826	0.927	0.933	0.913

ccp_alpha = 0.0005

tree number	20	40	60	100	200
depth=8	0.837	0.769	0.769	0.913	0.910
depth=12	0.848	0.826	0.929	0.933	0.911
depth=16	0.848	0.826	0.927	0.933	0.913
depth=20	0.848	0.826	0.927	0.933	0.913
depth=24	0.848	0.826	0.927	0.933	0.913
depth=28	0.848	0.826	0.927	0.933	0.913
depth=32	0.848	0.826	0.927	0.933	0.913

ccp_alpha = 0.001

tree number	20	40	60	100	200
depth=8	0.837	0.769	0.769	0.913	0.910
depth=12	0.848	0.826	0.929	0.933	0.911
depth=16	0.848	0.826	0.927	0.933	0.913
depth=20	0.848	0.826	0.927	0.933	0.913
depth=24	0.848	0.826	0.927	0.933	0.913
depth=28	0.848	0.826	0.927	0.933	0.913
depth=32	0.848	0.826	0.927	0.933	0.913

ccp_alpha = 0.002

tree number	20	40	60	100	200
depth=8	0.774	0.610	0.622	0.798	0.923
depth=12	0.798	0.826	0.929	0.929	0.962
depth=16	0.798	0.826	0.929	0.929	0.937
depth=20	0.798	0.826	0.929	0.929	0.937
depth=24	0.798	0.826	0.929	0.929	0.937
depth=28	0.798	0.826	0.929	0.929	0.937
depth=32	0.798	0.826	0.929	0.929	0.937

Precision value is average of 5 run with different random training set

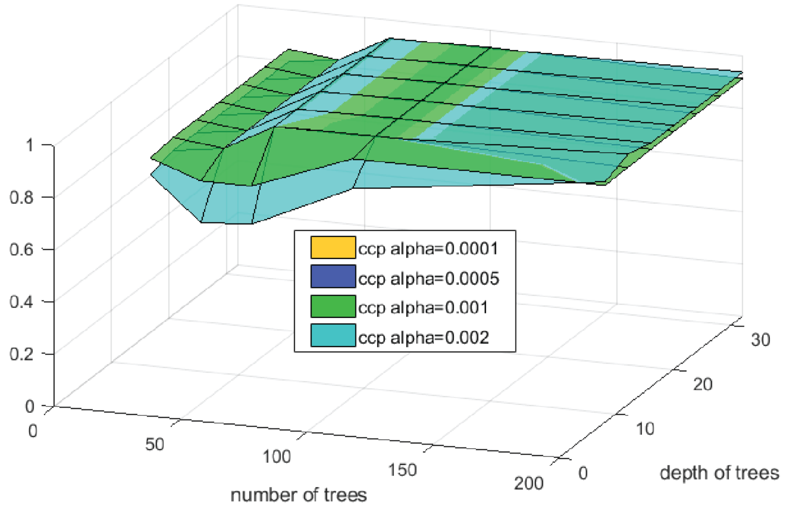

Fig. S6. Grid search results for the origami metasurface dataset. The table shows the mean precision of five separate runs for predicting the target class. This figure studies the influence of three hyper parameters: cost complexity pruning alpha (ccp_alpha), the number of trees, and the depth of trees. The cost-complexity pruning alpha value does not have a significant influence on the precision and the results for ‘ccp_alpha=0.0001’, ‘ccp_alpha=0.0005’, ‘ccp_alpha=0.001’ are overlaid on top of each other.

Origami Gripper (10Hz < frequency; heating power < 0.7W; maximum temperature < 500°C; 0.0015 N/m < stiffness)
Precision of kNN: 0.413; Precision of Boosting: 0.753

gini (minData=5)

tree number	20	40	60	100	200
depth=8	0.469	0.601	0.601	0.603	0.590
depth=12	0.710	0.863	0.842	0.842	0.807
depth=16	0.771	0.818	0.840	0.836	0.803
depth=20	0.830	0.837	0.849	0.836	0.803
depth=24	0.830	0.819	0.819	0.806	0.803
depth=28	0.830	0.819	0.819	0.806	0.803
depth=32	0.830	0.819	0.819	0.806	0.803

entropy

tree number	20	40	60	100	200
depth=8	0.796	0.816	0.843	0.829	0.846
depth=12	0.816	0.815	0.828	0.850	0.860
depth=16	0.816	0.815	0.828	0.844	0.874
depth=20	0.816	0.815	0.828	0.844	0.874
depth=24	0.816	0.815	0.828	0.844	0.874
depth=28	0.816	0.815	0.828	0.844	0.874
depth=32	0.816	0.815	0.828	0.844	0.874

Precision value is average of 5 run with different random training set

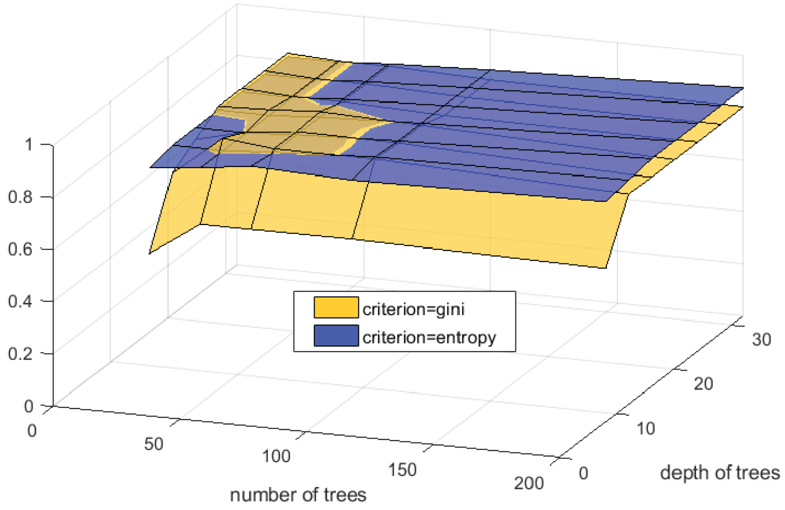

Fig. S7. Grid search results for the origami gripper dataset. The table shows the mean precision of five separate runs for predicting the target class. This figure studies the influence of three hyper parameters: tree branching criterion, the number of trees, and the depth of trees. The entropy criterion for selecting the splitting rule generally provides similar or better precision than using the Gini index.

Origami Arch (Zstiff > 800 N/m; Xstiff > 600 N/m; error < 0.1;)

Precision of kNN: 0.218; Precision of Boosting: 0.906

subset ratio: 50%

tree number	20	40	60	100	200
depth=8	0.898	0.915	0.915	0.935	0.872
depth=12	0.862	0.911	0.911	0.911	0.827
depth=16	0.862	0.911	0.911	0.911	0.805
depth=20	0.862	0.911	0.911	0.911	0.805
depth=24	0.862	0.911	0.911	0.911	0.805
depth=28	0.862	0.911	0.911	0.911	0.805
depth=32	0.862	0.911	0.911	0.911	0.805

subset ratio: 60%

tree number	20	40	60	100	200
depth=8	0.842	0.875	0.881	0.881	0.849
depth=12	0.888	0.843	0.814	0.814	0.815
depth=16	0.888	0.843	0.814	0.814	0.815
depth=20	0.888	0.843	0.814	0.814	0.815
depth=24	0.888	0.843	0.814	0.814	0.815
depth=28	0.888	0.843	0.814	0.814	0.815
depth=32	0.888	0.843	0.814	0.814	0.815

subset ratio: 70%

tree number	20	40	60	100	200
depth=8	0.895	0.832	0.832	0.843	0.826
depth=12	0.828	0.814	0.814	0.811	0.808
depth=16	0.828	0.814	0.814	0.822	0.808
depth=20	0.828	0.814	0.814	0.822	0.808
depth=24	0.828	0.814	0.814	0.822	0.808
depth=28	0.828	0.814	0.814	0.822	0.808
depth=32	0.828	0.814	0.814	0.822	0.808

subset ratio: 80%

tree number	20	40	60	100	200
depth=8	0.861	0.905	0.856	0.896	0.905
depth=12	0.843	0.830	0.867	0.836	0.843
depth=16	0.843	0.830	0.877	0.836	0.843
depth=20	0.843	0.830	0.877	0.836	0.843
depth=24	0.843	0.830	0.877	0.836	0.843
depth=28	0.843	0.830	0.877	0.836	0.843
depth=32	0.843	0.830	0.877	0.836	0.843

Precision value is average of 5 run with different random training set

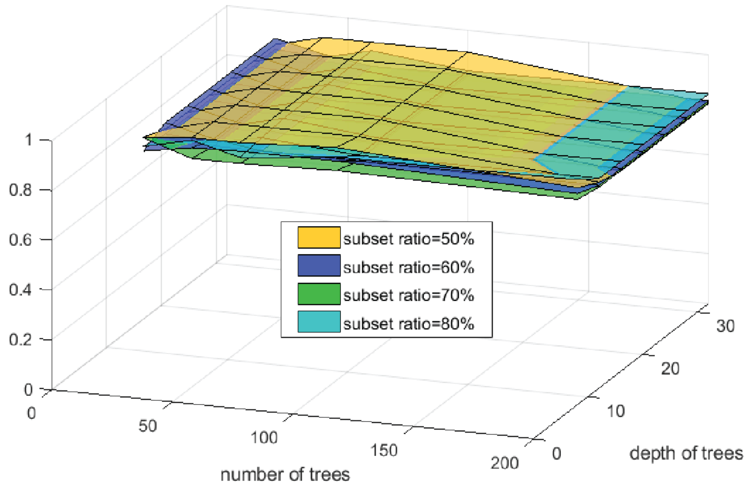

Fig. S8. Grid search results for the origami arch dataset. The table shows the mean precision of five separate runs for predicting the target class. This figure studies the influence of three hyper parameters: subset ratio, the number of trees, and the depth of trees. The subset ratio can affect the performance of the algorithm, and the results show that in general a smaller number can give better results.

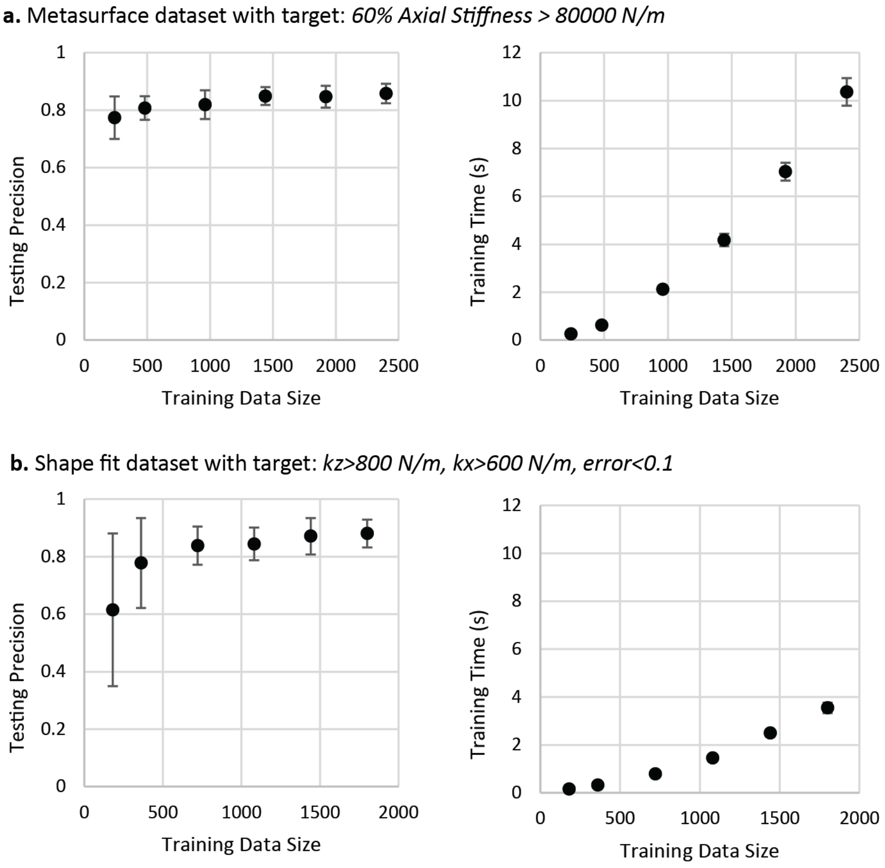

Fig. S9. Influence of data size on testing precision and training time. The figure shows that the testing precision increases and then saturates when using larger training dataset. The training time also increases as the data size get larger.

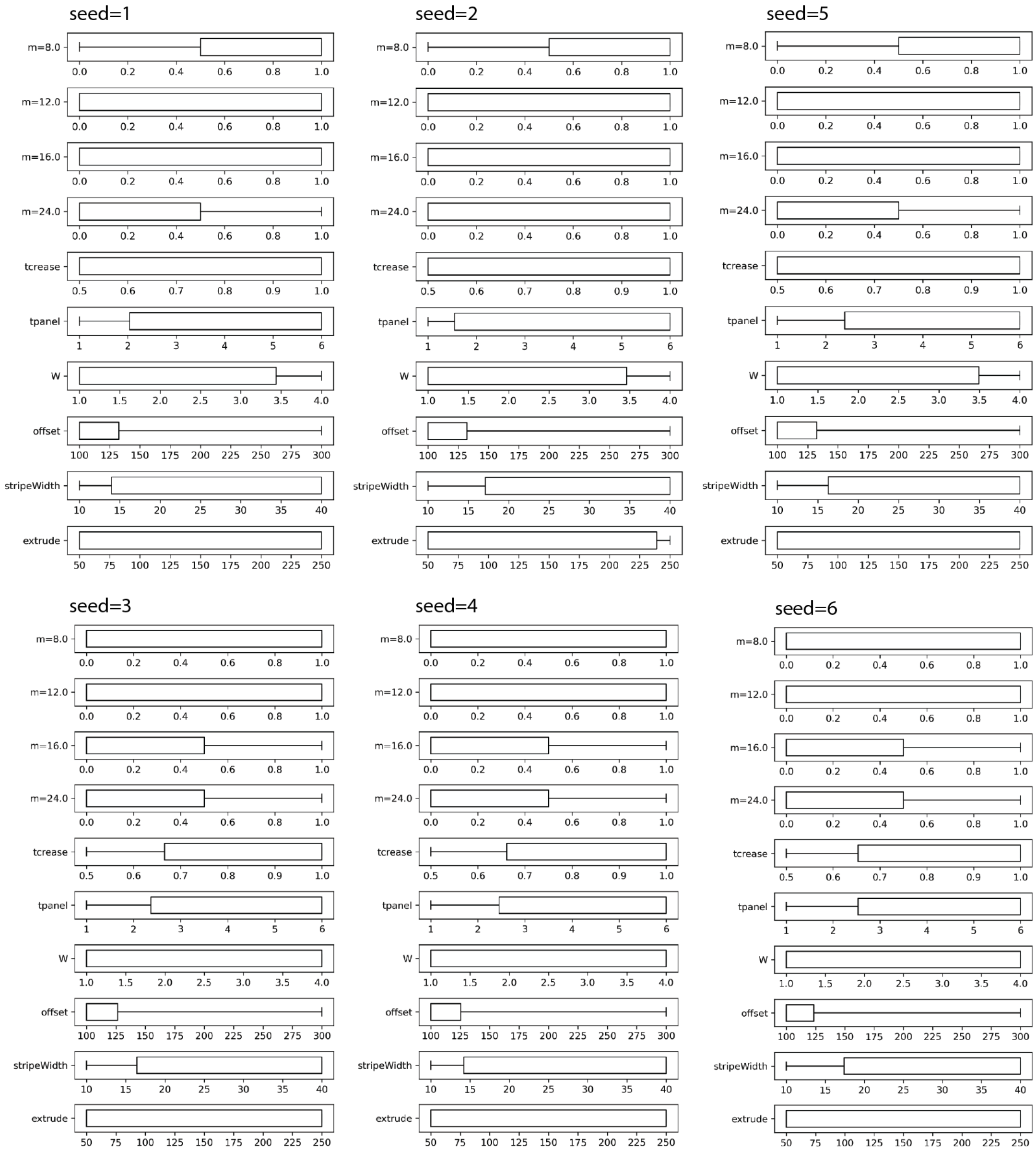

Fig. S10. Stable inverse design can be obtained from the proposed method. When searching for an origami arch that has a snap-through behavior, the proposed method can consistently find two clusters of potential solutions despite using different training/testing data partition.

Table S1. Prediction precision of different ML method applied on different design target of the origami metasurface dataset

test scenario	kNN	decision tree-random forest	gradient boosting
1	0.67	0.86	0.93
2	0.41	0.74	0.72
3	0.41	0.74	0.74
4	0.45	0.8	0.82
5	0.66	0.76	0.71
6	0.72	0.89	0.83
7	0.64	0.73	0.54
8	0.92	0.92	1
**average**	0.61	0.805	0.786

Data S1 to S4. (Separate files)

Three origami performance databases are published as supplementary material of this work. These three databases are:

- **Data S1:** Database on single Miura origami cell
- **Data S2:** Database on origami canopy
- **Data S3:** Database on active origami gripper
- **Data S4:** Database on origami arch

All three databases can be found in the electronic supplementary material associated with this manuscript or through the following GitHub website:

- https://github.com/zzhuyii/GenerateOrigamiDataSet

Codes S1 to S2. (Separate files)

Two sets of codes are published as supplementary material of this work. These two sets of codes are:

- **Codes S1** for simulating origami performances to populate the databases (Matlab)
- **Codes S2** for computing the decision rules using the decision tree algorithms (Python)

These two sets of codes can be found on GitHub using the following links:

- https://github.com/zzhuyii/GenerateOrigamiDataSet
- https://github.com/zzhuyii/TreeForOrigami

References

[1] K. Liu and G. H. Paulino, "Nonlinear mechanics of non-rigid origami: an efficient computational approach," *Proc. R. Soc. A,* vol. 473, p. 20170348, 2017.

[2] E. T. Filipov, K. Liu, T. Tachi, M. Schenk and G. Paulino, "Bar and hinge models for scalable analysis of origami," *Int. J. Solids Struct.,* vol. 124, pp. 26-45, 2017.

[3] Y. Zhu and E. T. Filipov, "SWOMPS (Sequentially Working Origami Multi-Physics Simulator)," vol. (Online), p. https://github.com/zzhuyii/OrigamiSimulator, 2021.

[4] Y. Zhu and E. T. Filipov, "A bar and hinge model fro simulating bistability in origami structures with compliant creases," *Journal of Mechanisms and Robotics,* vol. 12, p. 021110, 2020.

[5] Y. Zhu and E. T. Filipov, "Rapid Multi-Physics Simulation for Electro-Thermal Origami Systems," *International Journal of Mechanical Sciences,* Vols. 202-203, p. 106537, 2021.

[6] S. E. Leon, G. H. Paulino, A. Pereira, I. F. M. Menezes and E. N. Lages, "A Unified Library of Nonlinear Solution Schemes," *Appl. Mech. Rev.,* vol. 64, p. 040803, 2011.

[7] Y. Zhu, M. Birla, K. R. Oldham and E. T. Filipov, "Elastically and Plastically Foldable Electrothermal Micro-Origami for Controllable and Rapid Shape Morphing," *Adv. Funct. Mater.,* vol. 30, p. 20003741, 2020.

[8] L. H. Dudte, E. Vouga, T. Tachi and L. Mahadevan, "Programming curvature using origami tessellations," *nature materials,* vol. 15, pp. 583-588, 2016.

[9] C. Rudin, "Stop explaining black box machine learning models for high stakes decision and use interpretable models instead," *Nat. Mach. Intell.,* vol. 1, pp. 206-215, 2019.

[10] D. Bertsimas and J. Dunn, "Optimal Classification Trees," *Mach. Learn.,* vol. 106, pp. 1039-1082, 2017.

[11] L. Buitinck, G. Louppe, M. Blondel, F. Pedregosa, A. Mueller, O. Grisel, a. V. Niculae, a. P. Prettenhofer, A. Gramfort, J. Grobler, R. Layton, J. VanderPlas, A. Joly, B. Holt and G. Varoquaux, "API design for machine learning software: experiences from the scikit-learn project," *ECML PKDD Workshop: Languages for Data Mining and Machine Learning,* pp. 108-122, 2013.

[12] L. Breiman, J. Friedman, R. Olshen and C. Stone, Classification and regression trees, Monterey CA: Wadsworth and Brooks, 1984.
